# Defined enzyme cocktail from the anaerobic fungus *Orpinomyces* sp. strain C1A effectively releases sugars from pretreated corn stover and switchgrass

**DOI:** 10.1038/srep29217

**Published:** 2016-07-06

**Authors:** Jessica M. Morrison, Mostafa S. Elshahed, Noha H. Youssef

**Affiliations:** 1 Department of Microbiology and Molecular Genetics, Oklahoma State University, Stillwater, OK, USA

## Abstract

The anaerobic fungus *Orpinomyces* strain C1A is capable of growth on various types of lignocellulosic substrates, and harbors an impressive reservoir of carbohydrate active enzymes (CAZymes). Using a minimum enzyme cocktail strategy, we constituted a four-component lignocellulolytic cocktail derived from highly transcribed C1A, and evaluated its efficacy against pretreated corn stover and switchgrass. Hydrolysis yields ranged between 65–77.4%, depending on the lignocellulosic substrate and pretreatment applied. Addition of a highly expressed anaerobic fungal swollenin improved hydrolysis yields by up to 7%. Compared to the commercial cocktail CTec2, these anaerobic fungal cocktails provided comparable or slightly lower hydrolysis yields. Further, the differences in efficacy between commercial and anaerobic cocktails were often only realized after extended (168 hr) incubations. Under certain conditions, the hydrolysis yields of the anaerobic fungal cocktail was slightly superior to that realized by CTec2. We attribute the observed high hydrolysis yields to the high specific activity and affinity of the individual enzymes of the cocktail, as well as the high level of synergy and multi-functionality observed in multiple components. Collectively, this effort provides a novel platform for constructing highly effective enzymes for biofuel production and represents the first lignocellulolytic enzyme cocktail created from anaerobic fungal enzymes.

Lignocellulosic biomass represents a renewable and sustainable resource for the production of biofuels and bio-based chemicals. The most common approach for lignocellulosic biomass utilization is biochemical conversion, where enzymes are utilized for breaking down structural polymers in plant biomass to sugars (saccharification), followed by the conversion of produced sugars to products using dedicated sugar metabolizers. Improving the efficiency of enzymatic saccharification has been an active area of research during the last decade, with efforts dedicated towards the discovery and characterization of novel saccharolytic enzymes^1^, understanding mechanistic and structural aspects of enzymes catalysis^2^, and developing formulation strategies to maximize sugar release^3^.

Collectively, these efforts have resulted in a marked improvement in reported plant polymers hydrolysis yields. For example, reported glucan hydrolysis yields on alkaline pretreated corn stover has improved from 54.1% using Accellerase 1000 to 84.0% using Accellerase 1500/XY, with a similar improvement from 30.0% to 76.7% for xylan hydrolysis yield^4^. Nevertheless, it is still widely acknowledged that the current cost of saccharification enzymes represents the biggest hurdle towards wide scale production of lignocellulosic biofuels^5^. The actual cost of commercial enzymes ($/unit activity, or $/kg enzyme preparation) is not publicly advertised and is often negotiated on a case-by-case basis. However, a recent study estimates costs as high as $1.47/gallon ethanol^5^.

While the composition of commercial enzymes cocktails are proprietary, all such preparations are reported to contain a wide array of core enzymes (i.e. targeting the backbone of a specific polymer), as well as accessory debranching (removing side chains) or mobilizing (breaking bonds between various plant polymers) enzymes, in addition to various non-catalytic proteins or chemical adjuncts and surfactants^2^,^4^. The presence of 80–200 different components within a mixture has been reported^2^,^4^. Since the exact composition and production procedures/costs are not available, it is impossible to conduct a systematic cost/yield analysis to evaluate the relative contribution of individual cocktail components to the overall hydrolysis yields and assess whether their inclusion is economically justifiable. Nevertheless, it is intuitive to propose that the inclusion of a large number of individual enzymes/non-catalytic proteins and additives represents a substantial contribution to the overall cost of production.

Many of the activities reported in such commercial cocktails mediate the hydrolysis of polymers that are present in exceedingly low levels in common lignocellulosic substrates e.g. mannan (0.3–0.4% in corn stover and switchgrass)^6^ and pectin (2–10% in corn stover and switchgrass)^7^. Furthermore, the economic justification for the inclusion of multiple accessory enzymes has recently come into question^1^,^3^,^4^,^8^, since commonly utilized plant biomass pretreatment approaches often achieve the same outcome. Based on these arguments, we concur with Meyers *et al*.^3^ that the construction and optimization of a defined, highly effective, and minimal component enzymatic cocktail that solely targets the key abundant polymers (cellulose and hemicellulose) in lignocellulosic biomass represents an extremely promising approach for the development of a highly effective and lower cost lignocellulosic enzyme cocktail.

Currently, the majority of commercial enzymes are derived from aerobic fungi (e.g. *Trichoderma* and *Aspergillus* spp.) due to the relative ease of production and high yield in industrial settings^1^. Members of the anaerobic gut fungi (Phylum Neocallimastigomycota) represent an extremely promising and largely unexplored source of lignocellulolytic enzymes. Anaerobic fungi are residents of the rumen and alimentary tract of herbivores, where their growth and survival in these highly competitive systems is dependent upon their ability to metabolize ingested plant material in a fast and efficient manner^9^. We have previously argued that this constant evolutionary pressure is responsible for their observed remarkable biomass degradation potential and the acquisition of a rich CAZyme machinery^10^. Further, we reason that the relatively short plant biomass resident time within the herbivorous gut could represent an evolutionary driver for the selection of enzymes capable of the degradation of substituted/partly substituted hemicellulose polymers within their short span of availability. Prior studies on anaerobic fungal enzymes are relatively sparse, and have exclusively focused on characterizing single enzyme activities rather than cocktail formulation^11^.

Due to the ecological and evolutionary considerations outlined above, we hypothesize that anaerobic fungal core lignocellulosic enzymes will show high specific activities, multi-functionalities, synergy, and will be able to extract sugars from lignocellulosic biomass in the absence of accessory enzymes. Here, we report on the identification, cloning, expression, and purification of the most highly expressed genes mediating hydrolysis of cellulose (endoglucanase, cellobiohydrolase/exoglucanase, and β-glucosidase) and xylan (xylanase and β-xylosidase), and used these enzymes to formulate enzyme cocktails that efficiently hydrolyzed the cellulose and xylan components in both corn stover and switchgrass. The high hydrolysis yields obtained, coupled with the simplicity and defined nature of these preparations, renders them a promising alternative to commercial preparations and highlights the value of bioprospecting for novel enzymes in poorly studied lignocellulolytic microorganisms.

## Results and Discussion

### Cloned enzymes

We used a transcriptomics-guided strategy to identify carbohydrate-active enzyme (CAZyme) transcripts that are highly expressed by C1A when grown on lignocellulosic biomass substrates as candidates for cloning, expression, and characterization (Table 1). The sequence identity of chosen proteins to their closest non-Neocallimastigomycota sequences in GenBank database ranged between 37% (BGL1) to 71% (Bgxg1). As well, several of the cloned enzymes displayed even lower sequence identity to their closest biochemically-characterized relatives Bgxg1 (26%, Table 1).

### Substrate specificity, kinetics, and physiological characterization

We cloned, expressed, and characterized six novel lignocellulolytic enzymes and characterized their efficacy, substrate-specificity, and physiological optima. The enzymes obtained exhibited strong exoglucanase/cellobiohydrolase (Cel6A), endoglucanase (EG5), β-glucosidase and β-xylosidase (Bgxg1)^12^, and xylanase (XYL11) activities (Table 2). These enzymes were also found to have extremely high substrate affinity as evident by their extremely low *K*_*m*_ values (Table 2).

All enzymes displayed strong (top 33^rd^ percentile, Tables S2–S7) primary activity similar to those predicted from sequence homology data (EG5 – endoglucanase, Cel6A and Cel48 – cellobiohydrolase and exoglucanase, BGL1 and BGL3 – β-glucosidase, XYL11 - xylanase, Table 2). In addition, several enzymes were found to be multifunctional, and exhibited relaxed substrate specificities. EG5 exhibited additional moderate xylanase and weak β-glucosidase activities (Table 2). This multi-functionality is quite common in GH5-family enzymes^13^. Cel48 exhibited an additional weak endoglucanase activity (Table 2). BGL3 showed an additional weak β-xylosidase activity (Table 2). XYL11 showed an additional weak β-glucosidase activity (Table 2). In addition, we recently reported on a GH39-family enzyme, which exhibits a unique, yet strong triple oligosaccharide hydrolase (β-xylosidase, β-glucosidase, and β-galactosidase) activities^12^.

**Figure 1:**
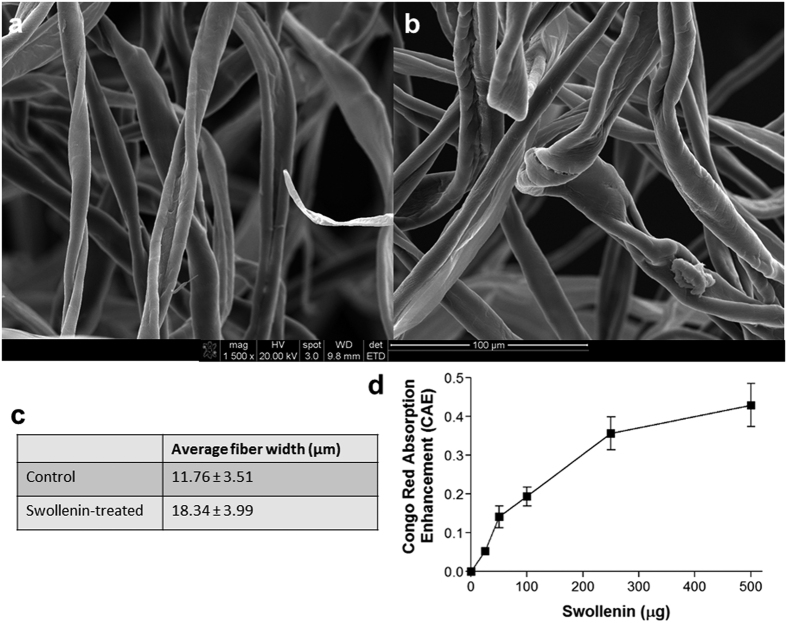
Swollenin activity testing. (**a**) Scanning Electron Microscopy of control cotton fibers. (**b)** Scanning electron microscopy of swollenin-treated cotton fibers. (**c)** Average cotton fiber width, calculated with ImageJ, using 10 random cotton fibers from each of 5 SEM images, measured at the widest point. Values shown are average ± standard deviation. (**d)** Congo red absorption enhancement (CAE) on swollenin-treated cotton at different concentrations, as described in^14^. Error bars shown are the standard deviation of triplicate samples (n = 3).

The Swol protein only displayed its predicted Swollenin activity (Table 2). Swol caused cotton fibers to visibly swell, enlarge, and untwist, with a significant difference in fiber diameter observed between Swol-treated cotton fibers and untreated cotton fibers (18.34 ± 3.99 μm vs 11.76 ± 3.51 μm diameters, p-value < 0.0001, Fig. 1a–c). Further, using a recently proposed dye-based cotton fiber assay^14^, swollenin-treated cotton fibers were able to absorb more Congo red than untreated cotton fibers, resulting in a significant Congo red absorption enhancement coefficient (Fig. 1d). The values achieved with Swol are significantly higher than that previously reported for comparable amounts of swollenin from *Bacillus* sp. AY8^14^.

All enzymes displayed circumneutral pH (optima in the 6–8 range) and temperature (optima between 39–50 °C) preferences (Table 2, Fig. S1). pH and temperature ranges varied, with Cel6A, Cel48, EG5, and XYL11 exhibiting activity over a wide range of pH values (Cel6A: 4–10, Cel48: 4–9, EG5: 3–8, XYL11: 3–9) and temperatures (Cel6A and EG5: 4–50 °C, Cel48 and XYL11: 4–60 °C (Table 2, Fig. S2), while both β-glucosidases, showing a relatively lower range of pH (5–9 for BGL1 and 6–8 for BGL3), and BCL3 showing a relatively low upper temperature limit (39 °C) (Table 2, Fig. S2).

### Formulation of cellulolytic and xylanolytic enzyme mixture

**Figure 2:**
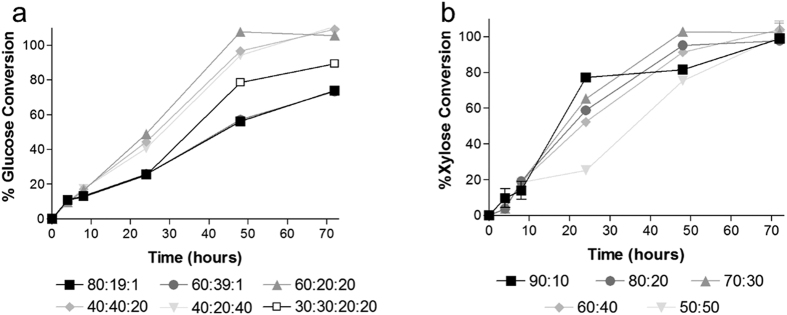
Initial enzymatic cocktail testing on Avicel and beechwood xylan using various cocktail formulation ratios. (**a**) Ratios of Cel6A:EG5:Bgxg1, or Cel6A:Cel48:EG5:Bgxg1 (30:30:20:20 only). (**b**) Ratios of XYL11:Bgxg1.

Based on activity measurements and physiological characterization of individual enzymes, we formulated a cellulolytic enzyme mixture containing EG5 (endoglucanase), Cel6A (possessing the highest cellobiohydrolase/exoglucanase activity), XYL11 (xylanase), and Bgxg1 (a multi-functional β-gluco-, xylo-, and galactosidase)^12^. We followed the rate and extent of Avicel degradation using mixtures with variable Cel6A:EG5:Bgxg1 ratios and a fixed overall protein amount (0.4 mg protein/1 g substrate (5% w/v), 20 μg/mL reaction). A ratio of 60:20:20 was deemed optimal, reaching 100% hydrolysis only 48 hours of incubation (Fig. 2a). In addition, we also examined whether the inclusion of Cel48 (a non-reducing end CBH1/2-type cellobiohydrolase) as a fourth component to complement Cel6A (a reducing end CBH1/2-type cellobiohydrolase) would improve Avicel degradation. By comparing the mixture of 60:20:20 (Cel6A:EG5:Bgxg1) to that of 30:30:20:20 (Cel6A:Cel48:EG5:Bgxg1), we show that only a single cellobiohydrolase/exoglucanase is needed and that cellulose degradation is actually better with only Cel6A in the mixture (Fig. 2a).

Similarly, we formulated a xylanolytic enzyme mixture containing various ratios of XYL11 (xylanase) and Bgxg1 (a multifunctional β-gluco-, β-xylo-, and β-galactosidase possessing the highest β-xylosidase activity)^12^. All cocktails reached 100% xylose conversion after 72 hours (Fig. 2b). The ratio 70:30 was deemed optimal, reaching 100% xylose conversion first, followed closely by 60:40 and 80:20 (Fig. 2b), which exhibited similar profiles.

### A four-component enzyme cocktail for biomass degradation

We subsequently evaluated the performance of a four-component enzyme cocktail (henceforth referred to as AGF4) for plant biomass degradation (Fig. S3). Our approach depends on utilizing a novel and relatively unexplored source of enzymes as a starting point. The lignocellulolytic abilities of anaerobic fungi are relatively well described^9^,^10^,^15^, and several prior studies have characterized highly active enzymes derived from the anaerobic fungi^12^,^16^,^17^,^18^. However, while their impressive lignocellulolytic repertoire is fairly well characterized, the anaerobic fungi remain relatively underutilized in biofuel-oriented research studies, and, to our knowledge, an enzymatic cocktail originating from anaerobic fungi has not been previously attempted. The anaerobic fungal enzymes described here are quire distinct from those derived from the aerobic fungi, *Trichoderma* and *Aspergillus* sp.^1^,^2^,^4^, that are thought to be the main components within commercial enzyme preparation. Further, in many cases, the enzymes characterized here are only closely related to genes identified in genomic sequencing studies and bear little similarity to all biochemically-characterized representatives within their CAZyme family. Preliminary evaluations suggested a 1:1 ratio (i.e. equal amount of both cellulolytic and hemicellulolytic cocktails) as optimal for biomass degradation (Fig. S3). The final AGF4 cocktail hence contained (Cel6A:EG5:Bgxg1:XYL11), with Bgxg1 acting simultaneously as the sole disaccharide hydrolase (for both cellobiose and xylobiose) in the mixture, at 30:10:25:35.

We tested the ability of AGF4 to release sugars from acid-, alkali-, and ionic liquid-pretreated corn stover and switchgrass. Pretreatment was an absolute necessity, as minimal sugar release (<0.3%) was observed with untreated corn stover and switchgrass using AGF cocktails (Fig. S4) and hence only pretreated (acid, alkaline, or ionic liquid) substrates are discussed in the main manuscript.

#### AGF4 yields

**Figure 3:**
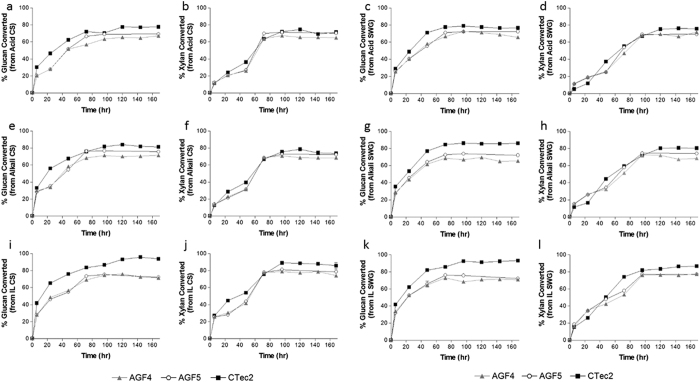
Enzymatic cocktail testing against commercial enzymes on various pretreatments of corn stover and switchgrass. (**a**,**b**) Acid-treated corn stover, (**c,d**) Acid-treated switchgrass, (**e,f**) Alkali-treated corn stover, (**g,h**) Alkali-treated switchgrass, (**i,j**) Ionic-liquid-treated corn stover, (**k,l**) Ionic-liquid-treated switchgrass. For (**a,c,e,g,i,k**) the percentage of glucan converted is shown. For (**b,d,f,h,j,l**) the percentage of xylan converted is shown.

On pretreated corn stover, AGF4 achieved final (t = 168 hr) glucan conversion yields ranging between 67.1% (acid) –71.5% (ionic liquid), and xylan yields ranging between 65.0% (acid) – 74% (ionic liquid) (Table 3). On pretreated switchgrass, AGF4 achieved final glucan conversion yields between 65.4% (alkali) – 70.8% (ionic liquid), and xylan yields between 68.5% (alkali) – 77.4% (ionic liquid) (Table 3). Within both plants, highest yields of glucan and xylan hydrolysis were observed with ionic liquid pretreatment, followed by alkali then acid pretreatments for corn stover and acid then alkali for switchgrass (Fig. 3, Table 3). The glucan yield on corn stover was significantly higher compared to switchgrass with alkali and acid pretreatments (p-value = 0.0426, Table 3), though conversion yields were similar with ionic liquid pretreatment on both substrates. In comparison, the xylan yield on switchgrass was significantly higher compared to corn stover with acid and ionic liquid pretreatments (p-value = 0.0178, Table 3), though conversion yields were similar with alkali pretreatment on both substrate.

#### Patterns of sugar release by AGF4

In general, glucan hydrolysis was significantly faster than xylan hydrolysis on all examined conditions regardless of substrate (corn stover or switchgrass) or pretreatment (acid, alkali, or ionic liquid) (Fig. 3, Table 3). By 72 hours, glucose released has consistently plateaued in all experiments, while xylose release steadily continued up to 96 hours (Fig. 3, Table 3). Indeed, the time needed to degrade 50% of glucan and xylan content (t_50_), as well as the time needed to metabolize 50% of the degraded proportion of glucan and xylan (t_50_deg) was significantly shorter for glucan (22–55 hours for corn stover, and 13–41 hours for switchgrass, depending on the pretreatment) compared to xylan (38–67 hours on corn stover and 38–70 hours in switchgrass, depending on the treatment) (Table 3, p < 0.0001 in all comparisons).

In general, glucan release (assessed by t_50_, t_50_deg values) was faster in switchgrass compared to corn stover (p-value < 0.0001 in all comparisons, Table 3) in all pretreatments. On the other hand, xylan release was faster in corn stover compared to switchgrass, although such difference was only significant for acid- and alkali-pretreatments (p-value = 0.0405 for acid, p-value = 0.0138 for alkali, Table 3). Glucan degradation rate (t_50_, t_50_deg) was significantly faster in ionic liquid pretreated biomass when compared to alkali-pretreated biomass (p-value = 0.015, Table 3) for both corn stover and switchgrass. In turn glucan degradation rate for alkali-pretreated biomass was significantly faster than that of acid pretreated biomass (p-value = 0.017) for both corn stover and switchgrass (Table 3). Xylan degradation rate (t_50_, t_50_deg) was significantly faster for both ionic liquid pretreated biomass and alkali-pretreated biomass when compared to acid-pretreated biomass for both corn stover and switchgrass (p-value = 0.032 for ionic liquid, p-value = 0.018 for alkali). However, the xylan degradation rate between ionic liquid pretreated biomass and alkali pretreated biomass was not significantly different on either corn stover or switchgrass (Table 3).

Collectively, these results demonstrate the feasibility and efficacy of a cocktail designed only from core lignocellulolytic enzymes that work to target the backbone of cellulose and xylan for the purpose of extracting the majority of sugars from pretreated plant biomass. The fact that AGF4, a core enzyme-only cocktail was incapable of hydrolyzing untreated biomass (less than 1% hydrolysis yields, compared to 30–60% glucan and xylan hydrolysis yields by CTec2 and Accellarase 1500/XY, Fig. S4) argues that pretreatment represents a viable substitute for some of the chemical transformations mediated by accessory components in commercial enzymes. Biomass pretreatments are known to increase the accessibility of enzymes to the cellulose and hemicellulose in pretreated biomass by increasing the surface area and porosity of plant biomass, by removing a significant amount of lignin, partially depolymerizing various hemicelluloses in the biomass, as well as reducing the crystallinity of cellulose^19^. Nevertheless, prior studies on the aerobic fungus *Aspergillus* compared core lignocellulolytic enzymes versus accessory enzymes and showed that only core enzymes (in their case) were not adequate at breaking down lignocellulosic biomass^1^. We hypothesize that, in addition to the pretreatment condition value, that the core enzymes in anaerobic fungi are better suited for the degradation of lignocellulosic biomass as compared to aerobic fungi. One can speculate that such success in anaerobic fungal enzymes could be explained by the transient and relatively short substrate resident time in the herbivorous gut, which provides evolutionary pressure that selects for enzymes capable of degrading immobilized polymers with intact or partially removed side chains.

### Swollenin addition improves glucan and xylan hydrolysis

We sought to determine whether the targeted addition of an accessory enzyme to AGF4 could enhance hydrolysis yields. As a proof of principle, we added Swol (0.8 mg/1 g biomass) and compared the performance of the five-component mixture (henceforth referred to as AGF5) to the AGF4 cocktail.

*AGF5 Yields.* An improvement in the final hydrolysis yield was obtained by Swol addition. The AGF5 cocktail could metabolize 69.3–73.6% glucan and 70.4–78.7% xylan in corn stover and 72.2–72.3% glucan and 69.4–77.0% xylan in switchgrass, depending on the pretreatment condition (Fig. 3, Table 3). Compared to AGF4, these values represent an overall improvement of 0.8–6.8% for glucan release, and 3.9–5.6% improvements for xylan release, when compared to AGF4 (Table 3). In both corn stover and switchgrass, improvements in glucan conversion was highest for alkali-pretreated substrates, followed by acid pretreated substrates, then by ionic liquid pretreated substrates (p-value = 0.0033, Table 3). On the other hand, the effect of Swol addition on xylan conversion differed between corn stover and switchgrass. For corn stover, the extent of improvement was highest with acid pretreatment, followed by ionic liquid pretreatment and then by alkali pretreatment (Table 3), although these results were not statistically significant (p-value = 0.88). For switchgrass, the only xylan conversion showing an improvement was the alkali pretreatment, whereas the acid and ionic liquid actually showed detrimental effects (Table 3).

#### Patterns of sugar release by AGF5

Time course analysis demonstrated that improvements in hydrolysis yields realized due to Swol addition (difference between grey triangle and white circles in Fig. 3, Table 3) is negligible within the first 24 hours, but are more pronounced between 72–96 hours and stays consistent to the experiment end. The inclusion of Swol resulted in faster glucan and xylan degradation, as evident by the shorter t_50_ and t_50_deg of AF5 versus AF4 in all experiments regardless of substrate or method of pretreatment. Significantly faster glucan conversion (t_50_) was seen for AGF5 as compared to AGF4 in both corn stover and switchgrass for acid and alkali pretreatments (p = 0.0486, Table 3), but not the ionic liquid pretreatment. In comparison, significantly faster xylan conversion (t_50_) was seen for AGF5 as compared to AGF4 for all pretreatment conditions, for both corn stover and switchgrass (p = 0.0093, Table 3).

### Comparison to commercial enzymes preparations

We compared the hydrolysis yields obtained by AGF4 and AGF5 cocktails to two commercial multicomponent enzyme cocktails, Accellerase 1500/XY^20^,^21^ and CTec2^22^. The final hydrolysis yields, as well as yields after 24, 72, and 168 hours of incubation were evaluated (Table 3). Initial studies identified CTec2 to be optimal at 3% enzyme cocktail loading and Accellerase 1500/XY to be optimal at 0.05 mL 1500 and 0.005 mL XY per 1 g biomass. Both Accellerase 1500/XY and CTec2 displayed very similar hydrolysis rates and yields (Fig. S5), and only comparison to CTec2 is presented here for simplicity.

#### Final yields comparison

Using CTec2, a final (t = 168 hr) glucan hydrolysis of 77.7–94.0% (corn stover) and 76.8–93.0% (switchgrass) were obtained (Table 3). Final xylan hydrolysis yields ranged between 71.6–85.9% on corn stover and 75.6–86.5% on switchgrass (Table 3). These final yields values are 3.8–22.3% higher than reciprocal values obtained with AGF4 and 1.3–21.7% higher than those obtained with AGF5 cocktails (Table 3). The difference in the final glucan conversion yields between CTec2 and AGF5 was least pronounced in alkali-pretreated corn stover (79.5% compared to 73.6% with AF5 for glucan and 73.7% compared to 72.3% for AF5 for xylan, Table 3), and was most pronounced in ionic liquid pretreatment, where CTec2 showed a remarkable >90% efficacy (Table 3, Fig. 3). In general, the differences were more pronounced in glucan when compared to xylan (p-value < 0.0001), and in corn stover as compared to switchgrass (p-value = 0.0924, Table 3, Fig. 3).

#### Patterns of sugar release comparison

Comparison of hydrolysis yields at 24, 72, and 168 hours between AGF4, AGF5 and CTec2 showed an interesting pattern (Table 3). During the initial 24 hours, CTec2 was more efficient in releasing of glucan in all pretreatments and substrates tested (Fig. 3, Table 3). This difference, however, was not observed with xylan conversion (Fig. 3, Table 3). By 72 hours, the gap in hydrolysis yields between CTec2 and AGF4/AGF5 significantly narrows (Fig. 3, Table 3). In fact, a relative efficiency comparison of total (glucan + xylan) hydrolysis yields demonstrates that AGF5 achieves similar, and sometimes superior efficiency to CTec2. This comparable relative hydrolysis ratio after 72 hours was observed in alkali-pretreated corn stover (1.01 relative efficiency), acid-pretreated corn stover (0.98 relative efficiency), ionic liquid-pretreated corn stover (0.93 relative efficiency), and acid-pretreated switchgrass (0.92 relative efficiency) (Table 3).

Final time point (t = 168 hr) yields comparison demonstrates that, on the whole, CTec2 appears to be more effective at converting residual glucan and xylan to the component sugars, as evident by the widening gap between it and AGF4/AGF5 between 72 and 168 hours (Fig. 3, Table 3). CTec2 was superior to AGF4/AGF5 under all conditions after 168 hours of incubation (Fig. 3, Table 3). While such results are understandable, given the magnitude of research efforts undertaken to formulate and optimize these cocktails, we believe that the AGF4/AGF5 and the relatively small difference in hydrolysis yields between AGF4/AGF5 and CTec2 are remarkable, given the minimal components and ease of formulation of the AGF cocktails. Our AGF5 cocktail was closest in performance to CTec2 in degrading glucan, compared to xylan, in corn stover rather than switchgrass, and after 72 hours of incubation, rather than any other time point (Table 3, Fig. 3). Indeed, under certain conditions, AGF5’s relative efficiency was slightly higher (in alkali-pretreated corn stover), or near equivalent (acid-pretreated corn stover, ionic liquid-pretreated corn stover, acid-pretreated switchgrass) to CTec2 (Table 3). Collectively, the results demonstrate that a defined cocktail from anaerobic fungi could provide biomass conversions comparable to the undefined and complex Accellerase 1500/XY and CTec2 cocktails, in spite of its overarching simplicity, especially in alkali pretreated substrates, in terms of xylan release, and in 72 hour incubation procedures.

The above results should be regarded as a proof of principle on the feasibility of constructing a defined, minimal component cocktail based on anaerobic fungal enzymes that could be used for efficient biomass conversion to sugars. The performance could be improved through the targeted addition of components, as well as formulation optimization. One tantalizing possibility that we are currently exploring is the combination of anaerobic fungal cocktails with copper oxidase cellulases (formerly GH61, now AA9 family CAZyme). These enzymes act by degrading cellulose in the presence of redox-active co-substrates under aerobic conditions and synergistically enhance cellulase activity, especially in recalcitrant areas of cellulose, and their discovery and inclusion in cocktails proved instrumental in improving yields in recent years^8^. It is interesting to note that both AGF4 and AGF5, whilst lacking AA9 (absent in the strictly anaerobic Neocallimastigomycota) are more efficient than all commercial enzymes reported prior to the discovery of AA9, and only slightly lower than currently available cocktails, all of which contain AA9 (Novozyme, personal communication).

## Conclusions

Using anaerobic fungal enzymes, we constituted a defined lignocellulolytic enzyme cocktail by cloning and expressing highly transcribed genes from *Orpinomyces* strain C1A. A minimal cocktail (4 proteins) was effective in saccharification of the majority of glucan/xylan content in corn stover/switchgrass with varying pretreatments. The addition of swollenin caused up to 7% enhancement in degradation of lignocellulosic biomass, from 69–79% hydrolysis, depending on pretreatment and biomass. Hydrolysis yields were slightly lower, but comparable to those from commercial cocktails (CTec2). The simplicity and availability renders this cocktail a promising starting point for enzyme cocktails instead of proprietary multicomponent enzyme preparations.

## Materials and Methods

### Transcriptomics-guided selection of lignocellulolytic enzymes

In a prior study, we examined the transcriptional response of strain *Orpinomyces* sp. strain C1A to growth on various types of lignocellulolytic substrates (alfalfa, energy cane, corn stover, and sorghum) as well as on glucose^23^. We identified various glycoside hydrolase (GH) families and transcripts mediating core activities necessary for cellulose (endoglucanases, cellobiohydrolases, and β-glucosidases) and xylan (xylanases and β-xylosidases) degradation. Using these datasets, we identified the highest expressed transcript mediating a specific-bioinformatically-predicted activity of those listed above (Table 1). In most cases, the chosen transcript was the highest expressed transcript mediating the predicted specific activity under all substrates examined. The choice of multiple annotated β-glucosidases is due to the multiple activities observed within members of GH1 and GH3 families, which often renders sequence-based predictions unreliable^24^. The choice of two annotated cellobiohydrolases was to test the perceived need for including both a non-reducing (GH6) and reducing (GH48) end acting cellobiohydrolase for efficient cellulose hydrolysis^8^.

### Gene synthesis, cloning, expression, and protein purification

Transcripts were codon optimized for expression in *E. coli* cells. The recombinant pET28a(+) plasmids *cel6a* (1283 bp), *cel48* (2538 bp), *eg5* (1253 bp), *bgl1* (2132 bp), *bgl3* (2574 bp), *xyl11* (1237 bp), and *swol* (1929 bp) were synthesized by GenScript (Piscataway, NJ) (Table 1). The plasmids contained *NdeI* and *XhoI* restriction sites flanking the inserts, as well as kanamycin resistance (*kan*) for selection purposes. Transformation, cloning and overexpression were conducted as previously described^12^. SDS-PAGE gels were ran to confirm expected protein sizes and purity (Fig. S6)^12^.

### Enzymatic activities and physiological characterization

Endo-1,4-β-D-glucanase (endoglucanase), exo-1,4-β-D-glucanase (exoglucanase), endo-1,4-β-xylanase (xylanase), and exo-β-D-mannanase (mannanase) activities were assayed using 3,5-dinitrosalicyclic-acid (DNS) as a detection agent using the following substrates: carboxymethyl cellulose sodium salt (CMC, 1.25% w/v), Avicel microcrystalline cellulose (1.25% w/v), beechwood xylan (1.25% w/v), and locust bean gum (0.5% w/v) respectively^12^. Assays were conducted for 2 hours in sodium acetate buffer (0.1 M).

Cellulose 1,4-β-cellobiosidase (cellobiohydrolase), 1,4-β-D-glucosidase (β-glucosidase), β-L-arabinosidase (arabinosidase), β-D-mannosidase (mannosidase), 1,4-β-D-galactosidase (β-galactosidase), acetylxylan esterase, and xylan-1,4-β-xylosidase (β-xylosidase) activities were determined by using *p-*nitrophenol-based (PNP) substrates (10 mM): *p*-nitrophenyl-β-d-cellobioside (PNPC,), *p*-nitrophenyl-β-d-glucopyranoside (PNPG), *p-*nitrophenyl-β-d-arabinofuranoside (PNPA), *p-*nitrophenyl-β-d-mannoside (PNPM), *p-*nitrophenyl-β-d-galactopyranoside (PNPGal), *p-*nitrophenyl-β-d-acetate (PNPAc), and *p*-nitrophenyl-β-d-xylopyranoside (PNPX), respectively^12^. Assays were conducted for 15 minutes in sodium acetate buffer (0.1 M), using sodium carbonate (1 M) as a stopping reagent. α-glucuronidase activity was tested using Megazyme α-glucuronidase assay kit (Wicklow, Ireland). CellG3 was used as an additional substrate for endo-1,4-β-D-glucanase (endoglucanase) activity, by use of the CellG3 Endo-Cellulase Assay Kit (Megazyme, Wicklow, Ireland). Based on specific activity values, enzymes were described as strong (top 33^rd^ percentile), moderate (middle 33^rd^ percentile), or week (bottom 33^rd^ percentile) compared to values reported in prior published studies.

Swollenin activity was determined by use of the Congo red cotton assay^14^. Cotton (100 mg) was treated for 12 hours with varying amount of Swol (0, 25, 50, 100, and 500 μg) at 39 °C in 10 mM Tris buffer (pH 8.2). After 12 hours, cotton was washed gently with water and dried at 30 °C for 2 hours. The treated cotton was placed into microfuge tubes with a solution of Congo red (0.1 mM Congo red in 10 mM Tris buffer, pH 8.2). The tubes were let incubate at room temperature for 2 hours. Following the incubation, the cotton was removed and the tube was centrifuged (6000× g, 1 minute) to remove residual cotton fibers. The optical density at 530 nm was measured, with the OD_530_ of the untreated cotton acting as the control. The OD_530_ difference between the control and Swol-treated samples is reported as the “Congo red adsorption enhancement coefficient,” as suggested previously^14^. In addition, Swol efficacy was evaluated by quantifying cotton fiber width using electron microscopy. This assay depends on the fact that swollenin non-hydrolytically causes swelling between cellulose fibers, without the production of reducing sugars^25^,^26^,^27^. Cotton samples were mounted on stubs with double-sided tape and gold-palladium-coated (Balzers Union MED 010 Au-Pd Sputter Coater, Liechtenstein). Samples were viewed with a Scanning Electron Microscope (FEI Quanta 600 field emission gun ESEM with Evex EDS and HKL EBSD). For each sample, (Swol-treated and control), five random images were taken at 1500 x magnification. Images were analyzed using ImageJ^28^. For each of the images, ten cotton fibers were analyzed for fiber width at their widest point to calculate an average cotton fiber width (and standard deviation) for the Swol-treated and control cotton fibers.

For each enzyme and substrate tested, a variety of enzyme concentrations were examined to determine the lowest amount of enzyme to use that yields the highest specific activity. This amount of enzyme was utilized in all characterization to follow: Cel6A (4.0 μg), Cel48 (8.4 μg), EG5 (0.7 μg), BGL1 (0.5 μg), BGL3 (32 μg), XYL11 (1.3 μg), and Swol (50 μg). All experiments were completed in triplicate. One unit (U) of enzymatic activity was defined as one μmol of product released (reducing sugars in DNS assays, PNP released in PNP substrate assays, aldouronic acid in α-glucuronidase assay, and 2-chloro-4-nitrophenol released in CellG3 assay) per minute. The specific activity was further calculated by determining the units of enzymatic activity per milligram of enzyme used in the assay. All enzymes were tested against all substrates listed above. *K*_*m*_ and *V*_*max*_values for all enzymes at all detected activities was determined using standard procedures^29^. The pH range and optima for all enzymes were determined by assaying the enzyme’s primary, annotated activity at a wide range of pH (4–10), and temperatures (25–60 °C) as described previously^12^. The pH stability of all enzymes was determined by assaying the primary, annotated activity of the enzyme following their exposure to a wide range of pH’s (3–13) for one-hour at 4 °C. The thermal stability for the enzymes was also determined in a similar manner following a one-hour long incubation at the following temperatures ranging from 4–70 °C. Relative specific activities in relation to the best performing condition (100% activity) are reported.

### Plant material, pretreatments, compositional analysis, and commercial enzymes

Mature Kanlow switchgrass (*Panicum virgatum* var. Kanlow) was obtained from the Oklahoma State University experimental plots in Stillwater, OK. Corn stover (*Zea mays*) was obtained from the Industrial Agricultural Products Center at the University of Nebraska in Lincoln. Samples were dried at 45 °C overnight and milled, as previously described^10^.

Alkali pretreatment was conducted by heating 4 g of dry plant material in 40 mL of 3% (w/v) NaOH solution inside a sealed serum bottle for 1 hour at 121 °C^10^,^30^. Acid pretreatment occurred by heating 4 g of dry plant material in 40 mL of 0.5% (v/v) sulfuric acid (H_2_SO_4_) solution inside a sealed serum bottle for 1 hour at 121 °C^10^,^31^. Ionic liquid pretreatment occurred by heating 5 g of dry plant material in 100 mL of 1-ethyl-3-methylimidazolium acetate ([C_2_mim][OAc], Sigma Aldrich), with stirring, inside a sealed serum bottle in an oil bath for 3 hours at 160 °C^32^,^33^. All of the pretreated materials were recovered by filtration, and washed with deionized water as previously described^30^,^31^,^32^,^33^. Pretreated materials were dried at 30 °C for approximately 48 hours prior to being used in the experiments described below. The amounts of cellulose and xylan in the different pretreated plant substrates was determined by using standard procedures for compositional analysis as described previously^34^. The composition of plant materials used in this study is shown in Table S1. All hydrolysis yields (% glucan conversion or % xylan conversion) were calculated as the percentage of actual sugar released based on the expected sugar concentration from the appropriate compositional analysis result (g sugar detected/g sugar expected from substrate, from Table S1).

### Enzymatic cocktail design and evaluation

**Figure 4:**
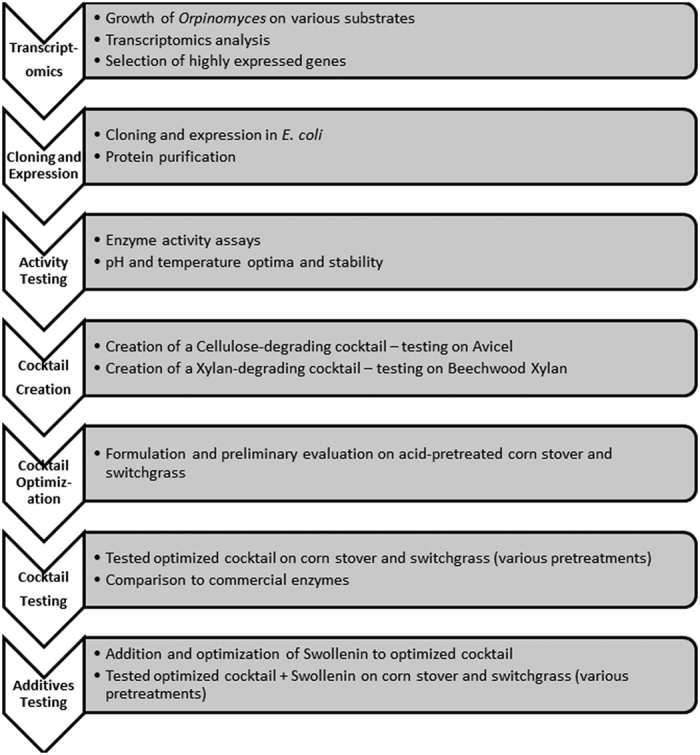
Schematic of experimental design.

We independently constituted and optimized two separate mixtures for cellulose (Avicel) and xylan (beechwood xylan) degradation. These two mixtures were subsequently pooled at various ratios, and evaluated on lignocellulosic substrates. The final cocktail was evaluated on acid, alkali, ionic liquid pretreated corn stover and switchgrass and compared to commercial enzyme preparations. An overall scheme of the process is presented in Fig. 4.

Experiments on Avicel and beechwood xylan were conducted using 70 μg enzyme with 0.175 mg substrate in 3.5 mL reaction volume (5% w/v substrate loading). A fixed protein concentration of 0.4 mg/g substrate (20 μg protein/mL reaction, 70 μg total protein for a 3.5 mL reaction) was used. Experiments were conducted in pH 6 buffer at 50 °C for 72 hours. The cellulolytic cocktail was constructed by mixing three enzymes: EG5 (GH5 endoglucanase), Cel6A (GH6 cellobiohydrolase/exoglucanase), and Bgxg1 (GH39 with dual β-glucosidase and dual β-xylosidase activity)^12^. Different ratios of Cel6A:EG5:Bgxg1 were tested (80:19:1, 60:39:1, 60:20:20, 40:40:20, 40:20:40). In addition, a four component cocktail was tested by adding a GH48 enzyme to the mixture to evaluate the inclusion of both reducing and non-reducing end cellulolytic enzymes (30:30:20:20, Cel6A:Cel48:EG5:Bgxg1). The xylanolytic cocktail was tested by mixing XYL11 (GH11 xylanase) and Bgxg1 (GH39 with dual β-glucosidase and dual β-xylosidase activity)^12^, at various proportions (XYL11:Bgxg1, 90:10, 80:20: 70:30, 60:40, 50:50). All experiments were conducted for 72 hours with samples taken at t = 0, 4, 8, 24, 48, and 72. At each time point, the samples taken were analyzed for glucose and xylose concentrations using PGO Enzyme Preparation Capsules (glucose) and the Megazyme Xylose Kit (xylose).

The enzymatic cocktail for cellulose and xylose degradation on lignocellulosic biomass was constructed and evaluated by mixing various proportions of the 3-component cellulolytic and the 2-component xylanolytic cocktails. The final cocktail (henceforth referred to AGF4) has 4 components due to the fact that Bgxg1 is present in both the cellulolytic and xylanolytic cocktails and has dual activity as a β-xylosidase and β-glucosidase^12^. The cellulolytic and hemicellulolytic cocktails were mixed and evaluated at a 50:50 ratio (corresponding to 30:10:25:35, Cel6A:EG5:Bgxg1:XYL11), 66:34 ratio (corresponding to 40:13:23:24, Cel6A:EG5:Bgxg1:XYL11), and 34:66 ratio (corresponding to 20:7:27:46, Cel6A:EG5:Bgxg1:XYL11). Preliminary optimization experiments were completed on acid-pretreated corn stover and switchgrass, with 0.8 mg of protein cocktail loaded per 1 g biomass and 5% w/v substrate loading. Experiments were conducted in pH 6 buffer at 50° C for 72 hours, with samples being taken at t = 0, 6, 24, 48, 72 hours. At each time point, glucose and xylose concentrations was assayed using PGO Enzyme Preparation Capsules (glucose) and the Megazyme Xylose Kit (xylose).

The AGF4 formulation resulting in the highest hydrolysis yields in the preliminary optimization was assessed on acid, alkali, and ionic-liquid pretreated corn stover and switchgrass, with 0.8 mg of protein loaded per 1 g biomass. In all experiments, dried biomass was used, with the exception of ionic liquid pretreated material, where a wet weight corresponding to 5% dry weight biomass loading was used to allow greater accessibility and prevent the dry biomass from absorbing the total liquid component as previously suggested^23^. Experiments were conducted at 50° C and pH 6 for 168 hours, with gentle agitation. Samples were taken at t = 0, 6, 24, 48, 72, 96, 120, 144, 168 hours. At each time point, the samples taken were analyzed for glucose and xylose concentrations using PGO Enzyme Preparation Capsules (glucose) and the Megazyme Xylose Kit (xylose).

For comparative purposes, Novozyme’s CTec2 (3% enzyme cocktail loading), and DuPont’s Accellerase 1500/XY (0.05 mL 1500 and 0.005 mL XY per 1 g biomass) were evaluated for their ability to breakdown untreated, alkali-treated, acid-treated, and ionic liquid-treated corn stover and switchgrass. The optimal conditions for these enzymes were determined, as suggested by the manufacturers: CTec2 was tested at 1.5%, 3%, 6%, and 30% enzyme cocktail loading^22^. As suggested by DuPont, Accellerase 1500 and Accellerase XY were mixed together in the following amounts: 0.05 mL 1500 and 0.005 mL XY per gram biomass, 0.1 mL 1500 and 0.01 mL XY per gram biomass, and 0.25 mL 1500 and 0.05 mL XY per gram biomass^20^,^21^.

Further, we investigated whether targeted addition of components into the system would aid in biomass reduction. We constituted a five-component enzyme cocktail (AGF5) by adding the non-catalytic swollenin (Swol) into the mix. The amount of swollenin needed was empirically determined by adding purified protein Swol to the AGF4 mixture at a concentration of 0.4 mg, 0.8 mg, 1.6 mg, 3.2 mg, and 6.4 mg per 1 g biomass (ratios of AGF4:Swol: 1:0.5, 1:1, 1:2, 1:4, 1:8, equivalent to 10 μg/mL, 20 μg/mL, 40 μg/mL, 80 μg/mL, and 160 μg/mL, respectively). Swol optimization was carried out using acid-treated corn stover (5% w/v) in pH 6 buffer at 50 °C for 96 hours, with samples taken at t = 0, 6, 24, 48, 96 hours. At each time point, the samples taken were analyzed for glucose and xylose concentrations using PGO Enzyme Preparation Capsules (glucose) and the Megazyme Xylose Kit (xylose). The best condition for AGF5 (0.8 mg cocktail loaded per 1 g biomass, and 0.8 mg Swol loaded per 1 gram biomass) was evaluated for its ability to breakdown all pretreated substrates as described previously. Experiments were conducted as described above, using 5% dry biomass loading (with the exception of the ionic liquid-treated material), pH 6.0 buffer, and a 168 hour reaction time at 50 °C with gentle agitation.

## Additional Information

**How to cite this article**: Morrison, J. M. *et al*. Defined enzyme cocktail from the anaerobic fungus *Orpinomyces* sp. strain C1A effectively releases sugars from pretreated corn stover and switchgrass. *Sci. Rep.* **6**, 29217; doi: 10.1038/srep29217 (2016).

## Supplementary Material

Supplementary Information

## Figures and Tables

**Table 1:** Enzyme cloned and expressed in this study.

Protein Name^1^	GH family	% transcript (GH family)^2^	Transcript length	CDS^3^	Protein length	Cloned region	Genbank Accession number^4^	Closest sequenced relative (% amino acid identity, accession number)^5^	Closest characterized Relative (% amino acid identity)^6^	Reference
EG5	5	7.7–30.9	1253	1–1251	417	All	KU963308	*Clostridiaceae* bacterium AN-C16-KBRB (52%, ADK66823)	*Clostridiaceae* bacterium AN-C16-KBRB (52%, ADK66823)	This study
Cel6A	6	60.4–83.7	1283	2–1177	391	All	KU963303	*Sorangium cellulosum* (45%, KYG0267)	*Aspergillus nidulans* FGSC A4 (46%, ABF50873.1)	This study
Cel48	48	1–30.3	2538	87–2342	751	All	KU963307	*Cystobacter fuscus* DSM2262 (54%, WP_002632298)	*Clostridium cellulolyticum* H10 (51%, ACL75108.1)	This study
XYL11	11	21.4–43.7	1237	3–1010	335	1–815	KU963309	*Fibrobacter succinogenes* subsp. *succinogenes* S85 (61%, AKN90969)	*Fibrobacter succinogenes* subsp. *succinogenes* S85 (61%, AKN90969)	This study
BGL1	1	27–79	2132	211–2130	639	250–2130	KU963306	*Eucalyptus grandis* (39%, XP_010044986)	*Phanerochaete chrysosporium* K-3 (37%, BAE87009.1)	This study
BGL3	3	4.5–40.9	2574	101–2380	759	164–2263	KU963305	*Mucor circinelloides* f. *circinelloides* 1006PhL (43%, EPB90789)	*Rhizomucor miehei* CAU432 (43%, AIY32164.1)	This study
Swol	NA^7^	21.1–52.3	1929	92–1822	576	182–1822	KU963310	*Aspergillus fumigatus* Z5 (56%, KMK55270)	*Trichoderma reesei* (56%)	This study
Bgxg1	39	58–84	1048	43–1048	335	109–1048	KT997999	*Clostridium saccharoperbutylacetonicum* (71%, WP_015392393)	*Caldicellulosiruptor saccharolyticus* (26%, AAB87373.1)	12

^1^Proteins used in the final AF cocktails are in bold.

^2^Values are from Couger *et al*., 2015, and refer to the percentage of transcripts within a specific GH family that are affiliated with the cloned transcript.

^3^CDS refers to the region in the mRNA that is transcribed. Numbering refers to the position in the mRNA itself.

^4^In addition to GenBank accession numbers (public release currently pending), the sequences of all enzymes are provided in the Supplementary document.

^5^Closest sequenced relative outside the Neocallimastigomycota.

^6^Closest sequenced and biochemically characterized relative outside the Neocallimastigomycota.

^7^NA: Not applicable.

**Table 2:** Substrate Specificity, Specific Activity, and Kinetics.

Enzyme	Substrate^a^	Activity Tested	Specific Activity (U/mg)	Specific Activity Percentile^b^	*K*_*m*_^c^	*V*_*max*_ (U/mg)	Temp. Range (°C)	Temp. Optima (°C)	pH range	pH optima
EG5	CellG3	Endoglucanase	209 ± 4.83	NA^d^	BDL^e^	775	4–50	50	3–8	7
	CMC	Endoglucanase	9.96 ± 0.24	48%	0.00027 mg/mL	622	4–50	50	3–8	7
	PNPG	β-glucosidase	0.29 ± 0.10	25%	84 mM	0.45	4–50	50	3–8	7
	Xylan	Xylanase	9.37 ± 1.17	44%	0.035 mg/mL	36.5	4–50	50	3–8	7
Not detected: Exoglucanase, Cellobiohydrolase, β-xylosidase, Arabinosidase, Acetyl Xylan Esterase, Mannosidase, β-galactosidase, Mannanase, or α-glucuronidase										
Cel6A	Avicel	Exoglucanase	2.03 ± 0.17	85%	0.00476 mg/mL	73.5	4–50	39	4–10	5
	PNPC	Cellobiohydrolase	2.82 ± 0.11	68%	0.228 mM	3.27	4–50	39	4–10	5
Not detected: Endoglucanase, β-glucosidase, Xylanase, β-xylosidase, Arabinosidase, Acetyl Xylan Esterase, Mannosidase, β-galactosidase, Mannanase, or α-glucuronidase										
Cel48	Avicel	Exoglucanase	0.20 ± 0.02	61%	1.257 mg/mL	0.218	4–60	39	4–9	5
	PNPC	Cellobiohydrolase	0.36 ± 0.03	40%	8.98 mM	0.357	4–60	39	4–9	5
	CellG3	Endoglucanase	0.04 ± 0.02	NA^d^	30.1 mM	0.048	4–60	39	4–9	5
Not detected: β-glucosidase, Xylanase, β-xylosidase, Arabinosidase, Acetyl Xylan Esterase, Mannosidase, β-galactosidase, Mannanase, or α-glucuronidase										
XYL11	Xylan	Xylanase	73.5 ± 6.18	75%	0.024 mg/mL	370	4–60	50	3–9	5
	PNPG	β-glucosidase	0.22 ± 0.01	18%	0.216 mM	0.28	4–60	50	3–9	5
Not detected: Exoglucanase, Cellobiohydrolase, Endoglucanase, β-xylosidase, Arabinosidase, Acetyl Xylan Esterase, Mannosidase, β-galactosidase, Mannanase, or α-glucuronidase										
BGL1	PNPG	β-glucosidase	1.09 ± 0.29	40%	0.164 mM	5.41	4–50	39	5–9	7
Not detected: Exoglucanase, Cellobiohydrolase, Endoglucanase, Xylanase, β-xylosidase, Arabinosidase, Acetyl Xylan Esterase, Mannosidase, β-galactosidase, Mannanase, or α-glucuronidase										
BGL3	PNPG	β-glucosidase	0.83 ± 0.10	36%	0.275 mM	1.55	4–39	39	6–8	6
	PNPX	β-xylosidase	0.06 ± 0.01	12%	50.6 mM	0.068	4–39	39	6–8	6
Not detected: Exoglucanase (Avicel), Cellobiohydrolase (PNPC), Endoglucanase (CellG3), Xylanase (Xylan), Arabinosidase (PNPA), Acetyl Xylan Esterase (PNPAc), Mannosidase (PNPM), β-galactosidase (PNPGal), Mannanase (Locust Bean Gum), or α-glucuronidase (Alduronic Acid)										
Bgxg1^f^	PNPG	β-glucosidase	73.4 ± 7.115	87%	BDL^e^	769	4–70	39	4–12	6
	PNPGal	β-galactosidase	54.6 ± 5.36	83%	BDL^e^	769	4–70	39	4–12	6
	PNPX	β-xylosidase	11.5 ± 1.2	51%	0.00485 mM	127	4–70	39	4–12	6
	Xylan	Xylanase	10.8 ± 1.25	43%	0.038 mg/mL	25.6	4–70	39	4–12	6
Not detected: Exoglucanase, Cellobiohydrolase, Endoglucanase, Arabinosidase, Acetyl Xylan Esterase, Mannosidase, Mannanase, or α-glucuronidase										
Swol										
Not detected: Exoglucanase, Cellobiohydrolase, Endoglucanase, β-xylosidase, β-glucosidase, Xylanase, Arabinosidase, Acetyl Xylan Esterase, Mannosidase, β-galactosidase, Mannanase, or α-glucuronidase										

“±” values represent the standard deviation of triplicate samples.

^a^Abbreviations: PNPC - *p*-nitrophenyl-β-d-cellobioside, PNPX - *p*-nitrophenyl-β-d-xylopyranoside, PNPG - *p*-nitrophenyl-β-d-glucopyranoside, PNPA - *p*-nitrophenyl-β-d-arabinofuranoside, PNPM - *p*-nitrophenyl-β-d-mannoside, PNPGal - *p*-nitrophenyl-β-d-galactopyranoside, PNPAc - *p*-nitrophenyl-β-d-acetate.

^b^Percentiles represent the percentage that the value is higher than (i.e. higher percentiles are better)

^c^*K*_*m*_ values are expressed in either mM or mg/mL, depending on the substrate tested. Values are shown ± standard deviation of triplicate samples (n = 3).

^d^NA: Not applicable. The reported specific activities for endoglucanases were reported using CMC, not CellG3 as a substrate, rendering activity comparisons inappropriate.

^e^BDL: Below detection limit (500 nM). Extrapolated *K*_*m*_ value obtained using Lineweaver-Burke plot was 0.000003999 mM (EG5-CellG3), 0.0000125 mM (Bgxg1-PNPG), 0.000214 mM (Bgxg1-PNPGal). Given the extinction coefficient of p-nitrophenol (PNP) is 17/mM/cm at 400 nm, for a 1 cm path length cuvette and absorbance minimum of 0.010, reliable Km detection limits in such PNP-based spectrophotometric assays is ≈ 500 nM. Therefore, Km values < 500 nM are referred to as BDL (below detection limit).

^f^Results shown for Bgxg1 are from^12^.

**Table 3:** Biomass degradation summary.

		Corn Stover			Switchgrass		
		Acid	Alkali	Ionic Liq.	Acid	Alkali	Ionic Liq.
AGF4	% glucan conversion	67.12 ± 0.95	69.53 ± 1.68	71.48 ± 0.93	65.79 ± 0.99	65.43 ± 1.27	70.76 ± 1.31
	% xylan conversion	65.03 ± 2.22	68.47 ± 0.45	74.30 ± 1.78	71.79 ± 0.78	68.53 ± 1.94	77.44 ± 1.71
	% total conversion	66.40 ± 0.79	69.24 ± 1.03	72.48 ± 1.23	66.88 ± 0.91	66.65 ± 1.33	73.83 ± 0.69
	t_50_ glucan (hours)	55	43	36	41	32	21
	t_50_deg glucan (hours)	33	27	22	23	19	13
	t_50_ xylan (hours)	67	59	56	70	63	52
	t_50_deg xylan (hours)	42	39	38	51	41	38
	Efficiency t = 24 h	0.65	0.65	0.73	0.87	0.95	0.98
	Efficiency t = 72 h	0.86	0.94	0.89	0.86	0.83	0.79
	Efficiency t = 168 h	0.88	0.89	0.80	0.88	0.80	0.82
AGF5	% glucan conversion	69.33 ± 1.80	73.57 ± 0.52	72.27 ± 1.38	72.24 ± 1.03	72.18 ± 2.12	72.33 ± 2.03
	% xylan conversion	70.35 ± 2.71	72.34 ± 0.43	78.68 ± 0.19	69.43 ± 2.62	74.13 ± 2.76	77.02 ± 1.03
	% total conversion	69.68 ± 0.76	73.26 ± 0.48	74.54 ± 0.92	71.88 ± 0.73	72.78 ± 0.72	74.48 ± 0.63
	t_50_ glucan (hours)	50	40	37	40	31	21
	t_50_deg glucan (hours)	32	29	23	27	21	14
	t_50_ xylan (hours)	63	59	54	67	60	47
	t_50_deg xylan (hours)	43	42	39	47	43	34
	Efficiency t = 24 h	0.65	0.65	0.69	0.88	0.97	0.97
	Efficiency t = 72 h	0.98	1.01	0.93	0.92	0.90	0.84
	Efficiency t = 168 h	0.92	0.95	0.82	0.94	0.87	0.83
CTec2	% glucan conversion	77.73 ± 1.42	79.51 ± 0.37	93.98 ± 1.60	76.77 ± 1.31	86.02 ± 1.18	93.03 ± 0.95
	% xylan conversion	71.64 ± 2.00	73.71 ± 1.76	85.89 ± 6.76	75.55 ± 1.31	80.49 ± 3.28	86.52 ± 2.78
	% total conversion	75.63 ± 1.32	77.49 ± 0.71	91.12 ± 3.43	76.45 ± 0.84	83.26 ± 0.78	90.04 ± 1.18
	t_50_ glucan (hours)	39	27	16	32	23	17
	t_50_deg glucan (hours)	26	21	15	23	20	15
	t_50_ xylan (hours)	62	53	38	65	59	48
	t_50_deg xylan (hours)	43	40	31	51	48	41

% glucan/xylan/total conversions shown were calculated at t = final (t = 168 hr), “±” values represent the standard deviation of triplicate samples (n = 3). Total conversion refers to the conversion of glucan and xylan combined. Relative efficiency was calculated at the noted time points from the % total conversion values and are calculated as compared to CTec2. t_50_ is calculated as the time needed to degrade 50% of the total glucan and xylan content and t_50_deg is the time needed to metabolize 50% of the degraded portion of glucan and xylan.
