# Supplementary material for: Defined enzyme cocktail from the anaerobic fungus Orpinomyces sp. strain C1A effectively releases sugars from pretreated corn stover and switchgrass
Source: Sci Rep. 2016 Jul 6;6:29217. doi: 10.1038/srep29217 (PMC4933900; doi:10.1038/srep29217)
Supplement: Supplementary Information [file srep29217-s1.pdf]

Supplemental Information

Defined enzyme cocktail from the anerobic fungus *Orpinomyces* sp. strain C1A effectively releases sugars from pretreated corn stover and switchgrass

Authors: Jessica M. Morrison[1], Mostafa S. Elshahed[1], and Noha H. Youssef[1*]

[1]Department of Microbiology and Molecular Genetics, Oklahoma State University, Stillwater, OK, USA

Supplementary File 1. Protein sequence of all enzymes cloned and expressed in this study.

>EG5
TLLGLAIIGTKAMRNISSKELVKELTIGWSLGNTLDATCFETLNYAKDQTASETCWGNV
KTTQELYYKLNDLGFNTFRIPTTWSGHFGDGPDYKISEAWMKRVHEIVDYALNADGYA
ILNIHHETWNHAFQKNLESAKKILVAIWKQIAAEFADYDEHLIFEGMNEPRKVGDAAEW
NGGDYEGWNFVNEMNDLFVKTIRATGGNNALRHLMIPTYAACVNDGAINNFRFPSGD
DKVIVSLHSYSPYNFALNNGAGAISNFNDGSEIDWVMNTINSSFISKGIPVIIGEFGAMNR
NNEADREKWAEYYIKKATSLGIPCVIWDNGYFEGEGERFGLIDRKSLNVVYPRLVNGL
MKGLGDKKPKTTKVRTTTTTTIQVQPTNNNDCFSTKLGYDCCNGCEVFYTDNDGKWG
VENGNWCG

>Cel6A
PKSSGGSTTTPVNPGDVDATPGDQLTLSGNPFSGVEFYINPYYVAEVDGAIAQMTDSSL
KAKAEKMKTYSNAIWLDTIKNMNSWLESNLKGALAQQTGSKKVLTVFVVYDLPGRDC
HALASNGELLANSSDAQRYKTEYIDVIEKHLKTYKSQPVALIVEPDSLANLVTNLESTPA
CRDSESYYLDGHAYLIKKLGVLPHVAMYLDIGHAFWLGWDDNREKAGKVYAKVIQSG
SPGKVRGFTDNVSNYTPWEDPTLSRGPDTEWNPCPDEKRYLEAMHKDFKAAGISSVYF
VSDTSRNGHKTDRKHPGEWCNQTGVGIGARPQASPISGMDYLDAFYWIKPLGESDGTS
DESAVRFDGYCGHETAMKPAPEAGQWFQKHFEQGLENANPPL

>Cel48
MPSIRTALTLLGASAVIAAPTKRANDAYAQRVIDTYNAVTGNGSYRSDYFSPENVPYHS
VETMMVEAPDQGHESVSETYSFWVWLEAINGKMTGNYDGVNTAWSYLEKHIIPDSKN
QPGMSRYNSSSPATYAPEDDDITNYPSKLIFQDGLVGQDPIAAELQQAYGTWDIYAMH
WIIDGDNWYGYGQQGDGTTKPSFINTFQRGPSESAWKTVPHPCWEEFKWGGRNGFLDL
FTVDNSYAKQWRYTAAPDADARAIQAAYFAYIWAKEDGQNLSSLAGKAAKLGDYLRY
AHYDKYFKKIGNCIGYQQCQAGSGKNSAHYLISWYFSWGGGLQGDWSWRIGSSHTHT
GYQNPLAAWVLSTESAFKPKGSTAVKDWATSLDRQLELFRWLQAAEGCIAGGATNSW
QGHYAQPPSDITSFYGMYYDWQPVYHDPPSNNWTGMQGWGMERVCSLYFLSGNEKA
GKVCQDWAKWVKNNVRVNGSEVVHATNFSWSGNPDEWKPANFNKSGLNASLHGTVS
TEGIDIGSLASIIKGLMWVSMKDGDQEGIDLAVQVMDAIEQYRDDHGYGVEEAREDYA
KFNEEVYIPSGWTGKNAQGANIRNGVKFIDIRPKYKQDPDWPQIENFLNGGEAPRFTYH
RFWAQTEIAVANGLISIYGLSDGSGVVPTKPGSCAAAIKAQGYKCCSANCEVVYTDADG
DWGVENDEWCGCGGGEPECPAAITNQGYPCCSTCGTVYYTDGDGDWGVENGDWCG
MPTDC

>XYL11
GSGTSGTADFPYAKVYIGNQQNQPSYQPTTTRNNSQPTSPATGGSSGEICSASGHSGEST
KVTSNKVGSINGIGYELWADSGNNSATFYSDGSFSCTFQNAKDYLCRSGLSFDSTKTHS
QIGHLYADFKLVKQGISNVDYSYVGIYGWTRSPLVEFYIVDNWLSQYRPGDWVGNKKH
GDFTIDGAQYTVYENTRTGPSIDGNTQFKQYFSIRKTARDCGTIDITAHFQQWEKLGMT
MGKMHEAKVLGEAGSNGSGTSGTADFPYAKVYTGSSSNNASSNNASSNNNNNNNNSSN
NNSSNSNCAARWAQCGGNGFNGAKCCKEGTCVQMNQWYSQCQ

>BGL1

FATLLAASAAKKCIVKPAADAAADVASDAEVVASDEENPLDNGEDSADDTPKGKLPSD
FKWGAATAAYQVEGAWNEEGRGESVWDHFTHLYPKNVESGDRSNKDSTNGNVACDS
YHKFDEDIKMMKIMNANHYRFSISWSRLFPDGQAKKVDGEWNVNEAGAQYYDMVIDT
LLDNDIIPLATLYHWDLPYALHEKYGGWLDYHSQFDFAKYAEFCFERYGDRVKGWITI
NEPWVNCVAGYKNGPGKAPYRCTGEAPRKLANDTTGLDLEGGCSYEIGPSQYTKDSEV
LPANRPPQKLEDVWCSHNILLGHAQAVKVYREKFQKEQKGLIGITIDGEAQIPWEEPGM
SEEEYENNKKYANLAAEFRIGWYSDPVMFGDYPESVKQRNGKDMPVFTDEEKKLLKGS
SSDFLGWNTYTSHWAAQVKNEDGSYVEPPTDEEASYDNSKRDMWDDNCKGRGDGWT
CIPPTLGSQAGSSWNTKYGPTIRVGLNWFSKRYEGIIKNGIIITENGCAQPNYKVSRANDQ
VTLDYWKSIGKEEFADTYDEDIIEDESDLEGTLIHDTYRIDWYKQYLENLRLAYVEDGV
DVRGYMAWSLVDNFEWENGYETRFGMTYIDFYNDKDLKRVPKDSLTFLGQWYLDNV
EQE

>BGL3
MKFSSVLSTVALLFVSKSLAITWEEADAKAREWCSDLTNEEKIALITGRENMTGVCVGSI
DPIERKGFKGLCLQDGPAGVRFAKGTATSWQASINTAATFDRTLLRKVGEAQGNEFYQ
RGINVALAPSMGIQRAPAAGRIFESYGEDPFYVGQCGLEVLKGIQSQGVIANAKHYIAND
QENNRGASTSNVPEQALWEVYMEPFYRTVVEGEAGTVMASYNAINGTYSTQNKRLLT
DYLKGKLGFKGFVMSDWWAIFDVEKSFGNGTDMNMPGGKYWGPDYVGDSFWGEHIQ
ECIDNGVFPQERLDDAVLRIIRTLYKLDQMDDYPEVNLYVDTNTKENAALNRKVGADS
NVLLKNADNILPIKKSKKVKKIAVIGKDSMPPTLCEDMKCANGTLPLGWGSGTTDFKYV
VDPLSAITKRAKKSKISVVSYGEDDAKKAAKVAKKADVAIVFVQADSGEEYITVEGNA
GDRLNLDLWHGGNELIEAVAAVNKNTIVVIHAPGPVNVPFLDKVKGIIFAGLPGQETGN
AITDVLFGDVNPSGHLPYTWAPREDYPTDVKYEPEYPDGGEKMTVYDYNEGLFVGYR
WFDKKEIEPTAFGYGLSYTTFEFSDLEATMEDDGLYVSLTVTNTGKVAGAAVPMIFLSF
PESVKYPIRLFKGFDKVMLKAGESKETTIFIDAHGLSYFDVEKMEYVRPKDGEFTVYAG
SNARDLPLNVKVLANGDEIDSDNDNSDEEINEDIEDVEDSSDDEDEKDLKKRFYKLY

>Swol
MKSIQALGLSLLIAGTYAKCQTGYECCKTCDVSYTDDDGEWGIEDGQWCGIDTEKCNK
SGDDTPVTSECWSKELGFDCCKSTTEVVAADASGKWGVENDQWCGIIESSATEDSCGA
YPCCKTCDSTYEDEDGKWAIENGDWCLLKKSCGDKPVDSTTTKATEPTTKSSGGVSRT
TLADGGILESGVFSTLPPSHTQPPYPHAENTGLASCGAKWTLVDNVCVAMYCEDDLSSE
NCDECGGVAGENGCVSVDPALCKSGIWPEVHDASDQPWKYSRSTHFGLTYGGACGFG
LYGLCTPKFNETGELCQRFCEHYPDLCADPEDISLRGNFCAPNGNYYTQFWSSLPGDYD
NYLSCGECYEVERTLPDGSSYPEGEKTDNIILQVLDSCPCTANAKWCCGSGTDHCNEIDF
KYGCPIPEESFHVDLSDIAMSRLQTNDPYGEMREGVIPIKYKRVPCPVKGNIHVWLRPGA
GPWYFALTVVNVANMGSLVAVEVQQADGKWVPLVRDPNYSSTRPQERYGSWTVKQG
TGPFETPMNMRFDSAFNTLTAENAIKDWGDPDFDMFDFLYIDTGVQFPMP

>Bgxg1
MKSLSLFFAYVLLVTPYLLVKATNVLTVECNNKLRRATHCANGSLYGITETTPRDYKSL
VDPLHPFVMRNPARGGNGNQHPYGDAIKVARRLADTPGALVSVDLPDMLPGWPYRWP
GMQSWLNQVKSFINDKKASGLKNWYGLEIWNEPDGTWNNSNGSFEEMWKQTYQVIR
QADPNEKIIGPCYSWYTDDKLRNFLKYAKANNCLPDIISWHELSGIDGVSSHLRSYREIE

KSLGIPELPISINEYCDAEHELEGQPGSSARFIGKFERYKVDTAMITWWFVPYPGRLGSLL
ATDTQKGAGWYFYKWYGDMTGDMLYVKPPNDNSKLVDGAACL

Table S1. Compositional analysis of pretreated corn stover and switchgrass.

Plant	Pretreatment	Glucan (%)	Hemicellulose (%)	Xylan (%)	Lignin (%)
Corn stover	Untreated	45	36	30	18
	Alkali	55	34	28	9
	Acid	42	25	22	33
	Ionic Liquid	31	26	17	43
Switchgrass	Untreated	40	38	30	23
	Alkali	48	34	30	18
	Acid	56	13	13	31
	Ionic Liquid	33	33	28	35

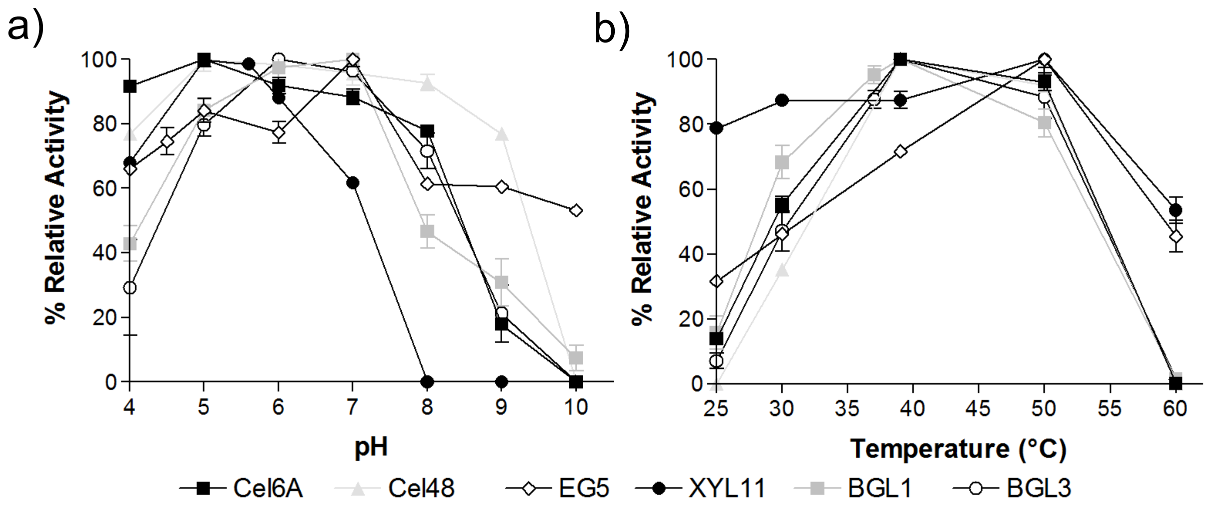

Figure S1. Optimal enzyme conditions. a) Optimal pH and b) Optimal Temperature. For all graphs, all values presented as relative specific activities. Error bars represent standard deviation of triplicate (n=3) samples.

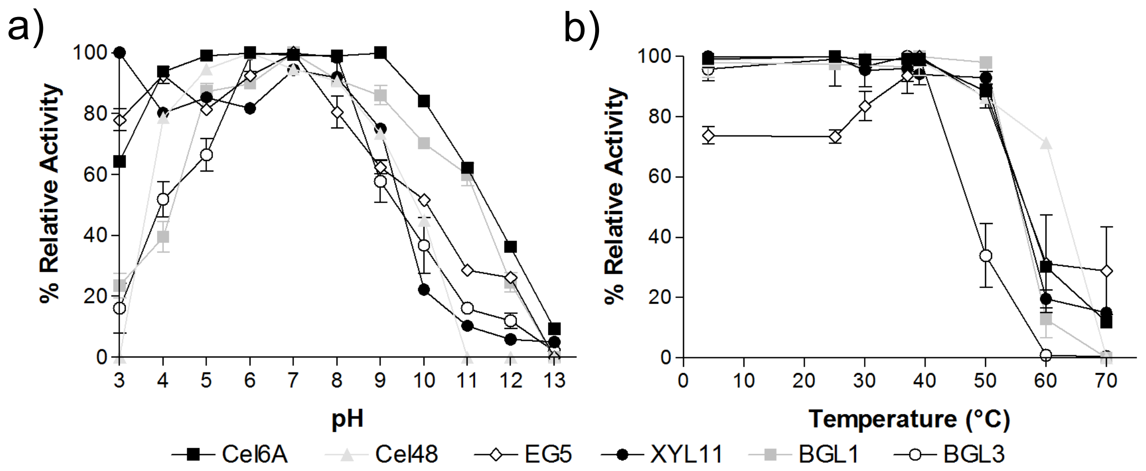

Figure S2. Enzyme stability. a) pH stability and b) thermal stability. For all graphs, all values presented as relative specific activities. Error bars represent standard deviation of triplicate (n=3) samples.

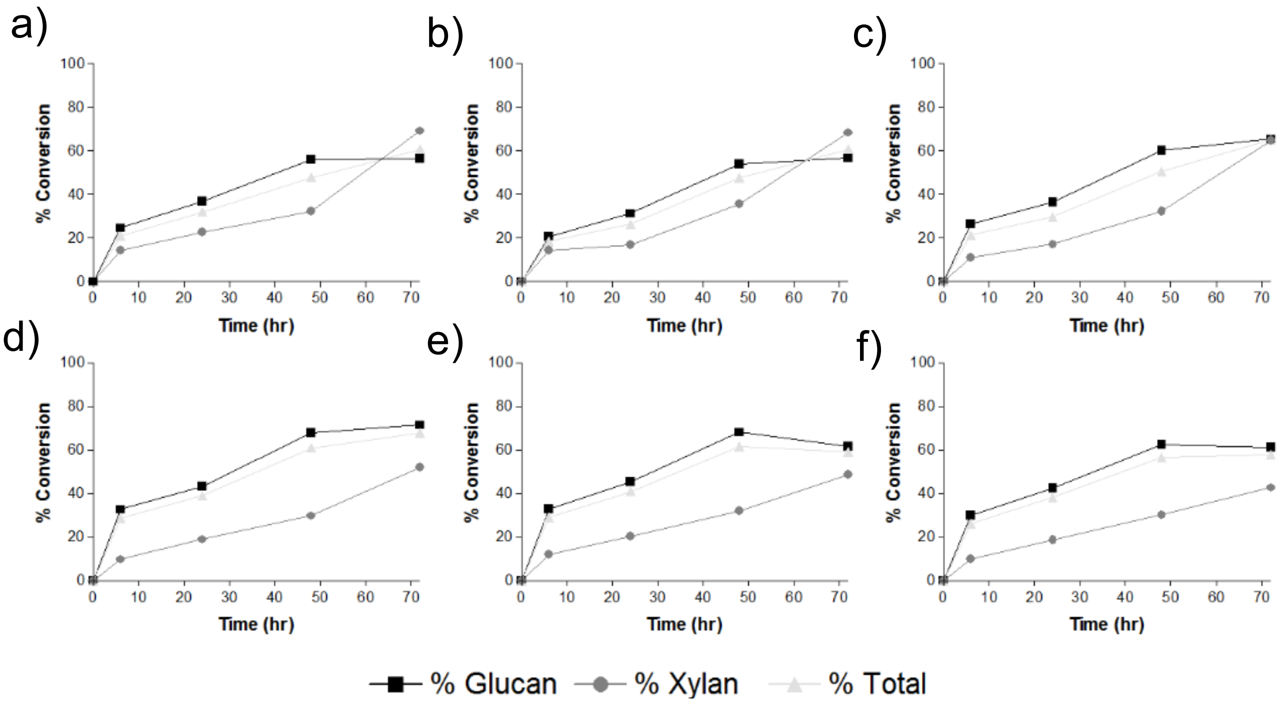

Figure S3. Enzymatic cocktail design examining ratios of cellulose-degrading cocktail and xylan-degrading cocktail on acid-pretreated corn stover (a-c) and switchgrass **(d-f).** Cel6A:EG5:Bgxg1:XYL11.ratios are as follows: **a** and **d**: 30:10:25:35; **b** and **e**: 40:13:23:24; and **c** and **f**: 20:7:27:46.

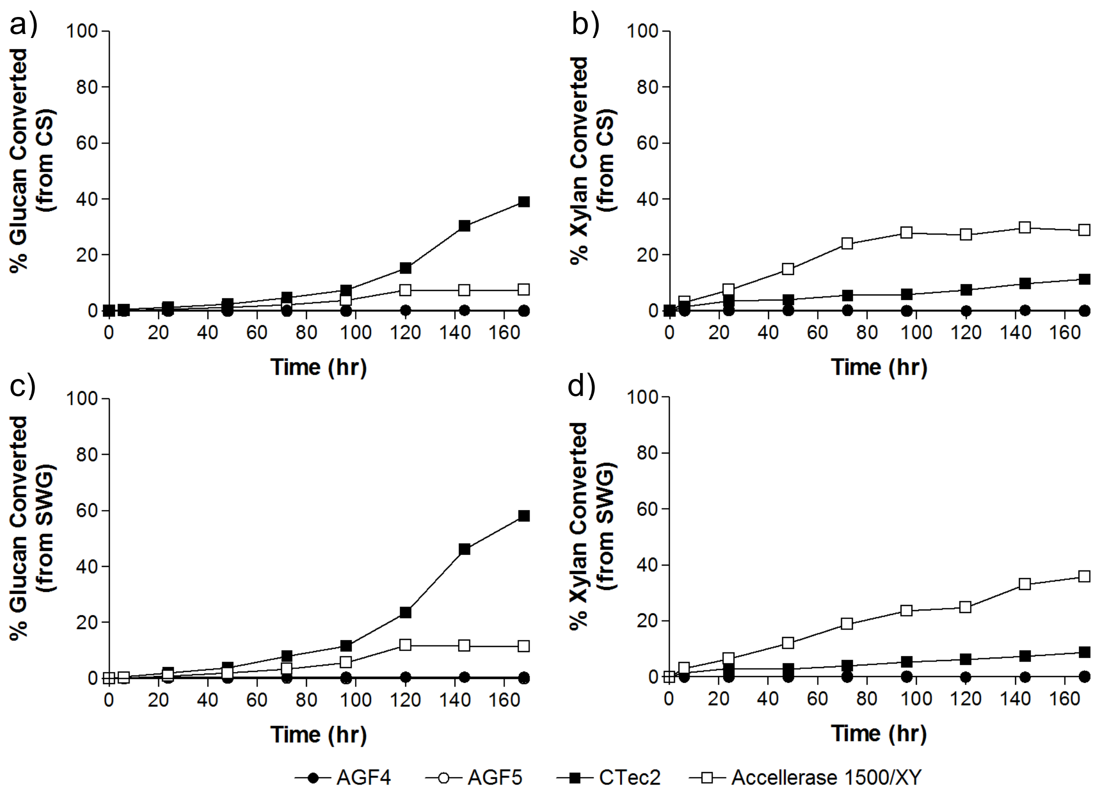

Figure S4. Enzymatic cocktails performance against untreated corn stover (a - glucan, b - xylan), and switchgrass (c - glucan, d - xylan).

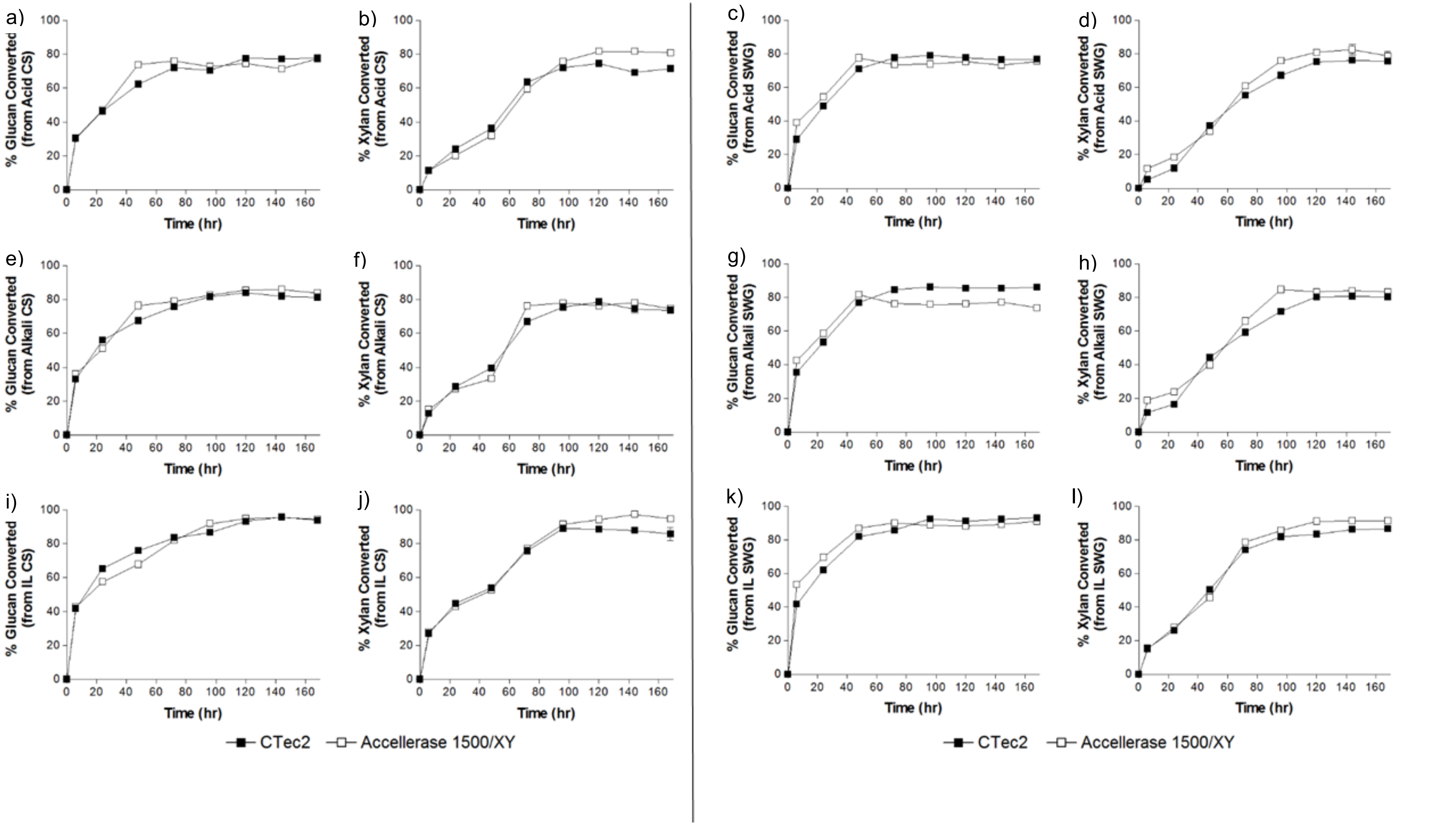

Figure S5. **Commercial enzyme preparations (CTec2 and Accellerase 1500/XY) tested on various pretreatments of corn stover (CS) and switchgrass (SWG). a-b)** Acid-treated corn stover, **c-d)** Acid-treated switchgrass, **e-f)** Alkali-treated corn stover, **g-h)** Alkali-treated switchgrass, **i-j)** Ionic-liquid-treated corn stover, **k-l)** Ionic-liquid-treated switchgrass. For **a, c, e, g, i, k,** the percentage of glucan converted is shown. For **b, d, f, h, j, l,** the percentage of xylan converted is shown.

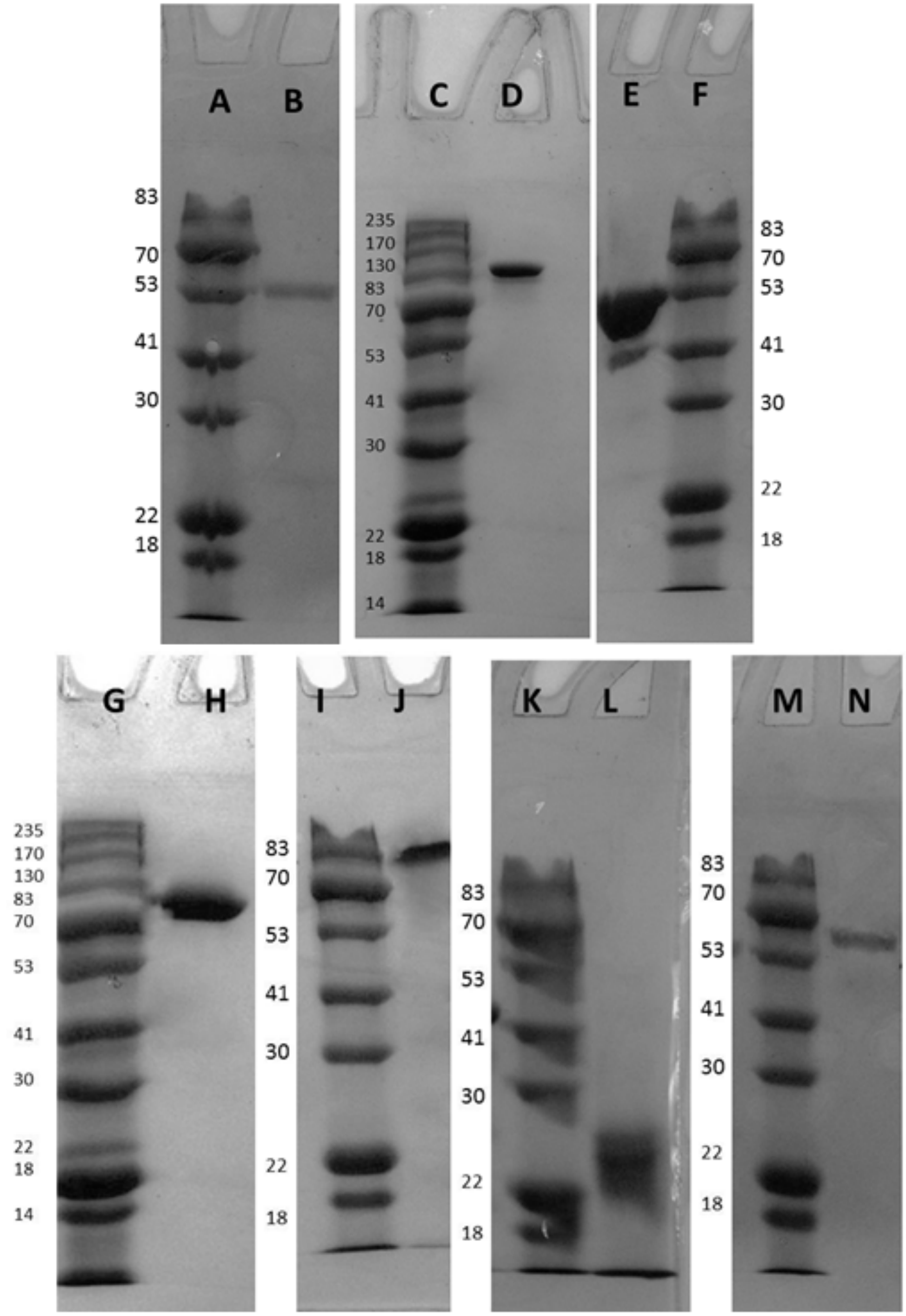

Figure S6. SDS-PAGE analysis of proteins. 12.5% SDS-PAGE analysis of recombinant proteins. *Lane A, C, F, G, I, K,* and *M*: Pre-stained Protein Ladder (Caisson Labs, Smithfield, Utah). *Lane B*: Cel6A, ~55 kDa; *Lane D*: Cel48, ~86 kDa; *Lane E*: EG5, ~49 kDa; *Lane H*: BGL1, ~75 kDa; *Lane J*: BGL3, ~80 kDa; *Lane L*: XYL11, ~25 kDa; *Lane N*: Swol, ~67 kDa. Protein molecular weights shown are consistent with the predicted protein size with linker and double-histidine tag.

Table S2. Properties of enzymes with exoglucanase activity All values listed are from enzymatic activities against Avicel. "-", not reported in the study. Rows of anaerobic fungi are highlighted.

Origin	Protein Name (Annotation)	Specific Activity (U/mg)	Kinetics Km (mg/mL)	Kinetics Vmax (U/mg)	Optimal pH	pH Stability	Optimal Temp. (°C)	Temp. Stability (°C)	Other Activities	Ref.
Orpinomyces sp. strain C1A	Cel6A	2.03	0.00476	73.5	5	4-10	39	4-50	Cellobiohydrolase	This Study
	Cel48	0.20	1.257	0.218	5	4-9	39	4-60	Cellobiohydrolase<br>Endoglucanase	This Study
Aspergillus nidulans	Exo-I	0.348	50	0.8	5.5	-	50	-	None	1
	Exo-II	0.785	38.4	1.3	-	-	-	-	None	1
	Exo-III	0.296	37.7	0.2	-	-	-	-	None	1
Chrysosporium lucknowense	EG25	0.05	-	-	4.5-6	5-8.5	60-70	60-75	Endoglucanase	2
	EG28	0.02	-	-	4.5-6	5-7	60-70	60-75	Endoglucanase<br>Xylanase	2
	EG44	0.06	-	-	4.5-6	5-7	60-70	60-75	Endoglucanase<br>Xylanase	2
	EG47	0.07	-	-	4.5-6	5-8.5	60-70	60-75	Endoglucanase<br>Xylanase	2
	EG51	0.19	-	-	4.5-6	5-7	60-70	60-75	Endoglucanase<br>Xylanase	2
	EG60	0.08	-	-	4.5-6	5-7	60-70	60-75	Endoglucanase<br>β-glucosidase	2
	CBHI	0.19	-	-	4.5-6	5-7	60-70	60-75	Cellobiohydrolase<br>Endoglucanase<br>β-glucosidase	2
	CBHII	0.08	-	-	4.5-6	5-7	60-70	60-75	Endoglucanase<br>Cellobiohydrolase	2
Fusarium oxysporum	Xylanase	0.02	-	-	7.4	6-8	50	to 50	Endoglucanase<br>Xylanase	3
Fusarium solani	Cellulase	1.34	-	-	5	5-7	65	to 65	β-glucosidase	4
Gloeophyllum trabeum	Cel5A	0.0045	-	-	-	-	-	-	Endoglucanase	5
	Xyn10A	0.0018	-	-	-	-	-	-	Xylanase	5
Humicola grisea	Exoglucanase I	0.00128	-	-	6-8	4-14	55-70	30-75	β-glucosidase<br>Endoglucanase<br>Cellobiohydrolase	6
Neocallimastix patricianum	CelA	1.1	-	-	6	5.2-11.3	50	to 70	Endoglucanase	7
	CelA	26.8	-	-	-	-	-	-	Endoglucanase	8
	CelB	1.4	-	-	-	-	-	-	Endoglucanase<br>Cellobiohydrolase	8
	CelC	0.9	-	-	-	-	-	-	Endoglucanase	8
	CelD	0.18	-	-	-	-	-	-	Endoglucanase	9

									Cellobiohydrolase	
Orpinomyces joyonii	CelA	0.12	-	-	4	3-7	40	to 50	Endoglucanase	10
Orpinomyces sp. PC-2	CelA	0.062	-	-	4.8	4.3-6.8	50	to 40	Endoglucanase	11
	CelC	0.113	-	-	5.6-6.2	4.6-7	40	to 40	Endoglucanase	11
Orpinomyces sp. Y102	CelC7	20.27	-	-	6.5	4-11	40	30-50	Endoglucanase	12
Penicillium brasilianum	EGa	0.04	-	-	-	-	-	-	Endoglucanase	13
	EGb1	0.02	-	-	-	-	-	-	Endoglucanase	13
	EGb2	0.06	-	-	-	-	-	-	Endoglucanase	13
	CBHa	0.11	-	-	-	-	-	-	Cellobiohydrolase	13
	CBHb	0.07	-	-	-	-	-	-	Cellobiohydrolase	13
Penicillium funiculosum	Cellulase	-	172	-	5	-	50	to 60	β-glucosidase<br>Cellobiohydrolase	14
Pichia pastoris	EXG1	1.07	3.5	192	-	-	-	-	β-glucosidase<br>β-xylosidase	15
Piromyces rhizinflatus	CbhYW23-2	6.2	-	-	6	-	46.4	to 50	Endoglucanase	16
Piromyces sp. E2	Cel6A	0.0053	-	-	-	-	-	-	Cellobiohydrolase	17
Termitomyces	Cellulase IF	24.6	-	-	4.4	3-5.5	55	to 42	Endoglucanase	18
Trichoderma harzianum	EXG	49.2	-	-	5.5	-	28	-	β-glucosidase<br>Endoglucanase	19
	Cellulase	130	-	-	4-5	-	37-40	to 40	None	20
Trichoderma neapolitana	CelB	0.71	-	-	6-6.6	-	106	to 106	Cellobiohydrolase	21
Trichoderma reesei	CBHII	0.001	-	-	-	-	-	-	Cellobiohydrolase	22
	EGI	0.4	-	-	-	-	-	-	Endoglucanase	22
	XYL9	0.00005	-	-	-	-	-	-	Xylanase	22
	XYL5.5	0.00005	-	-	-	-	-	-	Xylanase	22
	CBHI	0.0105	-	-	4	2-10	60	to 50	Cellobiohydrolase	23
	CBHII	0.0205	-	-	5	3-8	55	to 50	Cellobiohydrolase	23
	EGI	0.014	-	-	4	2-8	60	to 45	Endoglucanase<br>Xylanase<br>Cellobiohydrolase	23
	EGIII	0.0116	-	-	4	2-6	70	to 55	Endoglucanase	23
	CBH I	0.014	-	-	-	-	-	-	Cellobiohydrolase	24
	CBH II	0.027	-	-	-	-	-	-	Cellobiohydrolase	24
Trichoderma viride	Cel7B (EG I)	0.8	-	-	-	-	-	-	Cellobiohydrolase<br>Endoglucanase	25
	Cel2A (EG III)	0.4	-	-	-	-	-	-	Cellobiohydrolase<br>Endoglucanase	25
	Cel61A (EG IV)	0.3	-	-	-	-	-	-	Cellobiohydrolase<br>Endoglucanase	25
	Cel7A (CBH I)	0.3	-	-	-	-	-	-	Cellobiohydrolase<br>Endoglucanase	25
	Cel6A (CBH II)	0.7	-	-	-	-	-	-	Cellobiohydrolase<br>Endoglucanase	25

	Cel6B (CBH IIb)	0.2	-	-	-	-	-	-	Cellobiohydrolase<br>Endoglucanase	[25]
Anaerocellum thermophilum	CelA	0.055	-	-	5-6	-	85-95	-	Endoglucanase	[26]
Bacillus amyoliquefaciens	Cellulase	14	-	-	7	4-9	50	40-80	Endoglucanase<br>Cellobiohydrolase<br>Xylanase	[27]
Bacillus sp.	CH43	0.001	-	-	5-6.5	6-10	65	to 50	Endoglucanase	[28]
	HR68	0.0063	-	-	5-6.5	6-8	70	to 50	Endoglucanase	[28]
	E-H	1.1	-	-	-	-	-	-	Endoglucanase	[29]
	E-L	1.1	-	-	-	-	-	-	Endoglucanase	[29]
	Cel	0.014	-	-	-	-	-	-	Cellobiohydrolase	[30]
Bacillus sphaericus	Cellulase	5.76	-	-	8	7-10.5	60	to 70	Endoglucanase	[31]
Bacillus subtilis	Cellulase	2380	-	-	6	6-7.5	50-60	to 50	Endoglucanase	[32]
Caldibacillus cellulovorans	CMCase	1.7	-	-	6.5-7	-	80	to 70	Endoglucanase	[33]
Cellulomonas fimi	Cex	0.00016	-	-	-	6-9	-	-	Endoglucanase	[34-36]
	CenA	0.00218	-	-	-	-	-	-	Endoglucanase	[36]
	CenB	0.00222	-	-	-	-	-	-	Endoglucanase	[36]
	CenC	0.00099	-	-	-	-	-	-	Endoglucanase	[36]
	CenD	0.00242	-	-	-	-	-	-	Endoglucanase	[36]
Clostridium cellulolyticum	CelF	0.0134	2.4	-	5.5-6	-	55	20-45	Endoglucanase	[37]
Clostridium stercorarium	Avicelase II	0.041	-	-	5-6	4.5-7	75	65-80	None	[38]
Clostridium thermocellum	CelJ	0.0098	-	-	6	5-10	60	to 70	None	[39]
	CelS	0.019	-	-	5-6	-	70	-	None	[40]
	Endoglucanase	0.17	-	-	5.2	4.5-7	62	to 70	Endoglucanase	[41]
Pseudomonas fluorescens	Cellulase	6.9	5.3	10	6.5-7	5.8	35	to 50	None	[42]
Ruminococcus flavefaciens	Exo A	0.049	-	-	5	5-5.5	-	-	None	[43]
Streptomyces flavogriseus	Exoglucanase	0.019	-	-	-	-	-	-	None	[44]
Macrotermes mulleri	Cellulase IT	21	-	-	4.4	3-5.5	55	to 42	Endoglucanase	[18]
	Cellulase II	36.2	-	-	4.3-4.5	3-5.5	37	to 42	Endoglucanase	[18]

Table S3. Properties of enzymes with cellobiohydrolase activity. Table is organized by origin, fungal, then bacterial, then plant. All values listed are from enzymatic activities against *p*-nitrophenyl-β-D-cellobioside, PNPC. "-", not reported in the study. Rows of anaerobic fungi are highlighted.

Origin	Protein Name (Annotation)	Specific Activity (U/mg)	Kinetics Km (mM)	Kinetics Vmax (U/mg)	Optimal pH	pH Stability	Optimal Temp. (°C)	Temp. Stability (°C)	Other Activities	Ref.
Orpinomyces sp. strain C1A	Cel6A	2.82	0.228	3.27	5	4-10	39	4-50	Exoglucanase	This Study
	Cel48	0.36	8.98	0.357	5	4-9	39	4-60	Exoglucanase Endoglucanase	This Study
Acremonium persicinum	β-glucosidase	0.00075	0.91	-	5.5	-	-	-	β-glucosidase	[45]
Candida peltata	β-glucosidase	36.7	66	75	5	3.5-6	50	25-45	β-glucosidase	[46]
Chaetomium cellulolyticum	Xylanase 3	0.1	-	-	6-7	-	50	-	Xylanase Arabinosidase	[47]
Gloeophyllum trabeum	Cel5A	0.065	-	-	6-7	-	50	-	Endoglucanase Exoglucanase Xylanase	[5]
	Xyn10A	12	-	-	-	-	-	-	Endoglucanase Exoglucanase Xylanase	[5]
	Cel12A	0.069	-	-	-	-	-	-	Endoglucanase Exoglucanase Xylanase	[5]
Humicola grisea	Endoglucanase I	0.69	-	-	6-7	4-12	40-60	30-65	Exoglucanase Endoglucanase	[6]
	Endoglucanase II	0.8	-	-	5-6	6-10	55	30-55	Exoglucanase Endoglucanase	[6]
	Exoglucanase I	8.09	-	-	6-8	4-14	55-70	30-75	β-glucosidase Endoglucanase Exoglucanase	[6]
	β-glucosidase 1	0.91	-	-	4-5	4-12	60-65	30-60	β-glucosidase β-galactosidase β-xylosidase	[6]
	β-glucosidase 2	2.97	-	-	6-8	4-12	60-65	30-50	β-glucosidase β-galactosidase β-xylosidase	[6]
	β-glucosidase 3	3.19	-	-	6-8	4-12	60-65	30-50	β-glucosidase β-galactosidase β-xylosidase	[6]
	β-glucosidase 4	1.8	-	-	6-7	4-12	50-55	30-50	β-glucosidase β-galactosidase β-xylosidase	[6]
	β-glucosidase 5	3.45	-	-	6	4-12	60	30-55	β-glucosidase	[6]

								β-galactosidase<br>β-xylosidase	
	β-glucosidase 6	3.72	-	-	6-7	4-12	65-70	30-65	β-glucosidase<br>β-galactosidase<br>β-xylosidase	6
	BGL 4	1.8	-	-	6	6-11	55	to 50	β-glucosidase<br>β-galactosidase	48
Neocallimastix patricianum	CelB	1.31	-	-	-	-	-	-	Endoglucanase<br>Exoglucanase	8
	CelD	0.169	-	-	-	-	-	-	Endoglucanase<br>Exoglucanase	9
Orpinomyces joyonii	CelB2	4.9	-	-	5.5	5-7.5	40	to 50	Endoglucanase	49
Orpinomyces sp. PC-2	CelE	0.2	-	-	-	-	-	-	Endoglucanase	50
Paecilomyces thermophile	β-glucosidase	49.1	0.65	272	6.2	5-8.5	75	65-70	β-glucosidase<br>Exoglucanase	51
Penicillium brasilianum	EGa	0.34	-	-	-	-	-	-	Exoglucanase<br>Endoglucanase	13
	EGb1	0.38	-	-	-	-	-	-	Exoglucanase<br>Endoglucanase	13
	EGb2	1.16	-	-	-	-	-	-	Exoglucanase<br>Endoglucanase	13
	CBHa	2	-	-	-	-	-	-	Exoglucanase<br>Endoglucanase	13
	CBHb	0.98	-	-	-	-	-	-	Exoglucanase<br>Endoglucanase	13
Penicillium funiculosum	Cellobiohydrolase	0.56	-	-	4-5.5	2.5-5.5	60	to 70	Exoglucanase	52
	Cellulase	-	10	-	5	-	50	to 60	β-glucosidase<br>Exoglucanase	14
Phanerochaete chrysosporium	β-glucosidase	33	-	-	4-5.2	4-8	25	to 45	β-glucosidase	53
Piromyces equi	Cel5A	0.1	-	-	5.1	5.1-6.4	45	to 50	Endoglucanase	54
Sporotrichum thermophile	β-glucosidase I	1	0.83	-	5.4	4-6.5	65	to 65	β-glucosidase	55
Stachybotrys sp.	β-glucosidase p21	-	2.22	59	5	5-7	50	to 60	β-glucosidase	56
Trichoderma koningii	β-glucosidase	6.6	1.18	-	4-5	4-6	45	to 65	β-glucosidase	57
	β-glucosidase II	9.4	0.86	-	4-5	4-6	45	to 65	β-glucosidase	57
Trichoderma reesei	BGL 2	0.89	-	-	6	6-11	45	to 45	β-glucosidase<br>β-galactosidase	48
	CBH I	0.355	-	-	4	2-10	60	to 50	Endoglucanase<br>β-glucosidase	23
	EG I	9.19	-	-	4	2-8	60	to 45	β-glucosidase<br>Endoglucanase	23
	BGL I	0.894	-	-	4	5-9	70	to 55	β-glucosidase	23
	CBH I	0.00104	-	-	-	-	-	-	β-glucosidase<br>Endoglucanase	58
	CBH I	0.0103	-	-	-	-	-	-	Endoglucanase	59

									β-glucosidase	
	CBH II	0.0064	-	-	-	-	-	-	Endoglucanase<br>β-glucosidase	59
	EG I	0.133	-	-	-	-	-	-	Endoglucanase<br>Exoglucanase	60
	EG II	0.0075	-	-	-	-	-	-	Endoglucanase<br>Exoglucanase	60
	CBH I	0.46	-	-	-	-	-	-	Exoglucanase	60
	CBH II	0.048	-	-	-	-	-	-	Exoglucanase	60
Trichoderma viride	Cel7A (CBH I)	2.5	-	-	-	-	-	-	Exoglucanase	25
	Cel6A (CBH II)	5.7	-	-	-	-	-	-	Exoglucanase	25
	Cel6B (CBH IIb)	1.1	-	-	-	-	-	-	Exoglucanase	25
Bacillus amyoliquefaciens	Cellulase	24.6	-	-	7	4-9	50	40-80	Endoglucanase<br>Exoglucanase<br>Xylanase<br>β-glucosidase	27
Bacillus sp.	Cel	2.28	-	-	-	-	-	-	Exoglucanase	30
	E-H	0.3	-	-	-	-	-	-	Exoglucanase	29
	E-L	0.6	-	-	-	-	-	-	Exoglucanase	29
Cellulomonas fimi	Exg	3	0.641	-	6	5-9	60	60-75	Exoglucanase	61
Clostridium stercorarium	β-glucosidase	10.1	33	-	5.5	5-7	65	to 75	β-glucosidase<br>Exoglucanase	62
Clostridium thermocellum	β-glucosidase	33.3	-	-	6-6.5	5.5-7.5	60	to 68.5	β-glucosidase	63
	CelJ	0.0041	-	-	6	5-10	60	to 70	Exoglucanase	39
Ruminococcus flavefaciens	Exo A	0.298	-	-	5	4.5-5.5	-	-	Exoglucanase	43
Thermomonospora fusca	E1	0.04	-	-	-	-	-	-	Endoglucanase	58
	E5	0.0148	-	-	-	-	-	-	Endoglucanase	58
	E5cd	0.0144	-	-	-	-	-	-	Endoglucanase	58
	E6	0.00004	-	-	-	-	-	-	Endoglucanase	58
Thermatoga maritima	Cellulase I	0.035	-	-	6-7.5	-	95	to 80	Exoglucanase	64
	Cellulase II	0.015	-	-	6-7.5	-	95	to 80	Exoglucanase	64
	CelA	7.4	-	-	5	4-7	85-90	to 95	Exoglucanase	65
	CelB	0.05	-	-	7	5-9	85-90	to 95	Exoglucanase	65
Thermatoga neapolitana	CelA	69.2	0.97	69	6	-	95	to 95	Exoglucanase	21
	CelB	18.4	0.3	18	6-6.6	-	106	to 106	Exoglucanase	21

Table S4. Properties of enzymes with endoglucanase activity. Table is organized by origin, fungal, then bacterial, then plant. All values listed are from enzymatic activities carboxymethyl cellulose (CMC), unless otherwise noted. "-", not reported in the study. Rows of anaerobic fungi are highlighted. "*" value calculated with CellG3 as substrate.

Origin	Protein Name (Annotation)	Specific Activity (U/mg)	Kinetics Km (mg/mL)	Kinetics Vmax (U/mg)	Optimal pH	pH Stability	Optimal Temp. (°C)	Temp. Stability (°C)	Other Activities	Ref.
Orpinomyces sp. strain C1A	EG5	9.96<br>*209	0.00027<br>*0.000004	622<br>*775	7	3-8	50	4-50	β-glucosidase<br>Xylanase	This Study
	Cel48	*0.045	*30.1	*0.05	5	4-9	39	4-60	Exoglucanase<br>Cellobiohydrolase	This Study
Aspergillus aculeatus	XEG	-	3.6	260	3.4	3-3.8	30	to 50	None	66
Aspergillus nidulans	Endo-I	9.15	5	15	6	-	65	-	None	1
	Endo-II	8.26	4	12	5	-	50	-	None	1
Aspergillus niger	Cellulase	194	-	-	6-7	5-10	70	to 60	None	67
	Cellulase	45.2	14.5	85	3.8-4	1-9	45	to 25	None	68
	Cellulase	116.8	0.086	-	4	5-8	45-50	to 70	None	69
Chaetomonium cellulolyticum	Xylanase 1	0.06	-	-	6-7	-	50	-	Exoglucanase<br>Xylanase	47
	Xylanase 2	0.05	-	-	6-7	-	50	-	Exoglucanase<br>Xylanase	47
	Xylanase 3	0.03	-	-	6-7	-	50	-	Exoglucanase<br>Xylanase	47
Chrysosporium lucknowense	EG25	17	-	-	4.5-6	5-8.5	60-70	60-75	Exoglucanase	2
	EG28	11	-	-	4.5-6	5-7	60-70	60-75	Xylanase<br>Exoglucanase	2
	EG44	59	-	-	4.5-6	5-7	60-70	60-75	Xylanase<br>Exoglucanase	2
	EG47	14	-	-	4.5-6	5-8.5	60-70	60-75	Xylanase<br>Exoglucanase	2
	EG51	52	-	-	4.5-6	5-7	60-70	60-75	Xylanase<br>Exoglucanase	2
	EG60	12	-	-	4.5-6	5-7	60-70	60-75	Exoglucanase	2
	CBH I	0.2	-	-	4.5-6	5-7	60-70	60-75	Cellobiohydrolase<br>Exoglucanase	2
	CBH II	1.1	-	-	4.5-6	5-7	60-70	60-75	Cellobiohydrolase<br>Exoglucanase<br>Xylanase	2
Cryptococcus sp. S-2	CSCMCase	248.7	-	-	3.5	5.5-7.5	40-50	to 90	None	70
Fusarium oxysporum	Xylanase	5	13.1	-	7.4	5.8-8.2	50	to 50	Xylanase<br>Exoglucanase	3
Fusarium solani	Cellulase	4.75	-	-	5	5-7	65	to 65	β-glucosidase<br>Cellobiohydrolase<br>Exoglucanase	4

Gloeophyllum trabeum	Cel5A	120	-	-	-	-	-	-	Xylanase<br>Exoglucanase	5
	Xyn10A	0.95	-	-	-	-	-	-	Xylanase<br>Exoglucanase	5
	Cel12A	42	-	-	-	-	-	-	Xylanase<br>Exoglucanase	5
Humicola grisea	Endoglucanase 1	9.06	-	-	6-7	4-12	40-60	30-65	None	6
	Endoglucanase 2	24.1	-	-	5-6	6-10	55	30-55	None	6
Neocallimastix patricianum	CelA	108	-	-	6	5.2-11.3	50	to 70	Exoglucanase	7
	CelA	15.1	-	-	-	-	-	-	Exoglucanase	8
	CelB	53.6	-	-	-	-	-	-	Exoglucanase<br>Cellobiohydrolase	8
	CelC	20.6	-	-	-	-	-	-	Exoglucanase	8
	CelD	4.9	-	-	-	-	-	-	Exoglucanase<br>Cellobiohydrolase	9
Orpinomyces joyonii	Cellulase	203	0.5	-	5.5	5-7.5	40	to 50	Exoglucanase<br>Cellobiohydrolase<br>Xylanase	49
	CelB2	203	-	-	5.5	5-7.5	40	to 50	Cellobiohydrolase	49
	CelA	13.2	-	-	4	3-7	40	to 50	Exoglucanase	10
Orpinomyces sp. PC-2	CelE	3.2	-	-	-	-	-	-	Cellobiohydrolase	50
	CelB	2.9	-	-	-	-	-	-	None	50
	CelE	72.5	-	-	6	5.5-7.5	45	40-55	None	71
	CelA	1.2	-	-	4.8	4.3-6.8	50	to 40	Exoglucanase	11
	CelC	1.1	-	-	5.6-6.2	4.6-7	40	to 40	Exoglucanase	11
Orpinomyces sp. Y102	CelC7	25.96	-	-	6.5	4-11	40	30-50	Exoglucanase	12
Penicillium brasilianum	EGa	39.8	-	-	-	-	-	-	Exoglucanase	13
	EGb1	54	-	-	-	-	-	-	Exoglucanase	13
	EGb2	36.3	-	-	-	-	-	-	Exoglucanase	13
Penicillium funiculosum	Cellobiohydrolase	0.05	-	-	4-5.5	2.5-5.5	60	to 70	Cellobiohydrolase	52
Piromyces equi	Cel5A	3.2	-	-	5.1	5.1-6.4	45	to 50	Cellobiohydrolase	54
	Cel45A	42.5	-	-	6.5	5.2-7.9	60	to 65	None	54
Piromyces rhizinflatus	CbhYW23-2	6.0	-	-	6	-	46.4	to 50	Endoglucanase	16
Termitomyces sp.	Cellulase IF	378	-	-	4.4	2.8-5.5	55	to 42	Exoglucanase	18
Thermoascus aurantiacus	Cellulase I	0.525	3.9	6.3	5	6-8	75	to 65	None	72
	Cellulase II	0.0031	1.2	1.1	5	2-12	60	to 65	None	72
	Cellulase III	0.24	1.9	33	5	6-8	65	to 65	None	72
Trichoderma harzianum	Endoglucanase	312.5	-	-	5-5.5	-	40	to 35	None	20
	EG	0.63	-	-	5.5	-	-	-	None	19
Trichoderma reesei	CBHI	0.0021	-	-	-	-	-	-	Cellobiohydrolase<br>Exoglucanase<br>β-glucosidase<br>Xylanase	58

	CBHII	0.0014	-	-	-	-	-	-	Cellobiohydrolase<br>Exoglucanase<br>β-glucosidase<br>Xylanase	58
	CBHI	0.2	-	-	-	-	-	-	Cellobiohydrolase<br>Exoglucanase<br>β-glucosidase<br>Xylanase	59
	CBHII	21.5	-	-	-	-	-	-	Cellobiohydrolase<br>Exoglucanase<br>β-glucosidase<br>Xylanase	59
	EG I	59.8	-	-	4	2-8	60	to 45	Xylanase<br>Exoglucanase<br>β-glucosidase<br>β-xylosidase	23
	EG III	30.7	-	-	4	2-6	70	to 55	None	23
	EG V	9.22	-	-	4	2-6	50-65	to 80	None	23
Trichoderma viride	Cel7B (EG I)	7.6	-	-	-	-	-	-	Exoglucanase<br>Cellobiohydrolase	25
	Cel2A (EG III)	2.8	-	-	-	-	-	-	Exoglucanase<br>Cellobiohydrolase	25
	Cel61A (EG IV)	1.9	-	-	-	-	-	-	Exoglucanase<br>Cellobiohydrolase	25
	Cel6A (CBH II)	0.1	-	-	-	-	-	-	Exoglucanase<br>Cellobiohydrolase	25
	CMCase	0.258	0.88	3.5	4	3-7	50	30-70	None	73
	Cellulase	105	68	148	6.5	6.5-8	55	45-55	None	74
Anaerocellum thermophilum	CelA	1.758	-	-	5-6	-	85-95	-	Exoglucanase<br>Xylanase	26
Bacillus sp.	CH43	0.0062	1.5	0.93	5-6.5	6-10	65	to 50	Exoglucanase	28
	HR68	0.0159	1.7	1.7	5-6.5	6-8	70	to 50	Exoglucanase	28
	Cellulase	58.9	0.48	-	9	6-12	40	to 60	None	75
	Cellulase	49.4	11.4	135	8.6-9	5-11	45	to 60	β-glucosidase<br>Cellobiohydrolase<br>Exoglucanase<br>Xylanase	76
	E-H	23.5	-	-	-	-	-	-	Exoglucanase<br>Cellobiohydrolase	29
	E-L	43.8	-	-	-	-	-	-	Exoglucanase<br>Cellobiohydrolase	29
	Cellulase	71	-	-	10	6-10	50	to 60	None	77
	Cel	128	-	-	-	-	-	-	Cellobiohydrolase	30
	E-I	0.12	-	-	6	5-8	60	to 80	None	78

	E-II	60	-	-	7	7-10	50	to 60	None	78
	E-III	59	-	-	7	6-10	50	to 60	None	78
	Cellulase	246	-	-	6	5-7	50	to 50	None	79
Bacillus amyoliquefaciens	Cellulase	100	-	-	7	4-9	50	40-80	Exoglucanase	27
									Cellobiohydrolase	
Bacillus pumilus	Cellulase	0.079	-	-	6	5-9	60	30-70	Exoglucanase	80
									β-glucosidase	
Bacillus sphaericus	Cellulase	38.4	-	-	8	7-10.5	60	to 70	β-glucosidase	31
Bacillus subtilis	CelDR	0.3	-	-	6.5	-	50	to 75	None	81
	Cellulase	7001	-	-	6	6-7	50-60	to 50	Exoglucanase	32
Caldibacillus cellulovorans	CMCase	30.6	3.4	44.7	6.5-7	-	80	to 70	None	33
Cellulomonas fimi	Cex	0.01	-	-	-	6-9	-	-	Exoglucanase	34-36
	CenA	0.760	-	-	-	-	-	-	β-glucosidase	36
									Cellobiohydrolase	
									Exoglucanase	
	CenB	0.928	-	-	-	-	-	-	β-glucosidase	36
									Cellobiohydrolase	
									Exoglucanase	
	CenC	1.016	-	-	-	-	-	-	Exoglucanase	36
	CenD	0.01	-	-	-	-	-	-	Exoglucanase	36
	Exg	20	3.18	-	6	5-9	-	60-75	Exoglucanase	61
									Cellobiohydrolase	
Cellulomonas sp.	Cellulase I	162	-	-	-	-	-	-	None	82
	Cellulase CA	136	-	-	-	-	-	-	None	82
	Cellulase CB	9.7	-	-	-	-	-	-	None	82
Clostridium cellulolyticum	CelF	0.0014	-	-	5.5-6	-	55	20-45	Exoglucanase	37
Clostridium thermocellum	CelJ	13.1	4.06	164	6	5-10	60	to 70	Exoglucanase	39
									Cellobiohydrolase	
	Endoglucanase	65.1	-	-	5.2	4.5-7	62	to 70	Exoglucanase	41
Marinobacter sp.	CMCase	2548.75	-	-	9	4-12	27-35	-	None	83
Salinivibrio sp.	Cellulase	32.4	3.03	143	7.5	6.5-8.5	35	10-40	None	84
Sinorhizobium fredii	CMCase	3.822	-	-	7	6-9	35	30-45	None	85
Thermomonospora curvata	Cellulase	0.3	3.5	213	6	5-8	65	to 65	None	86
Thermomonospora fusca	E1	5.41	-	-	-	-	-	-	None	58
	E2	0.369	-	-	-	-	-	-	None	58
	E2cd	0.344	-	-	-	-	-	-	None	58
	E3	0.0013	-	-	-	-	-	-	None	58
	E4	0.122	-	-	-	-	-	-	None	58
	E5	2.840	-	-	-	-	-	-	None	58
	E5cd	2.480	-	-	-	-	-	-	None	58
	E6	0.0642	-	-	-	-	-	-	None	58
Thermatoga maritima	Cellulase I	1.2	-	-	6-7.5	-	95	to 80	Exoglucanase	64
									Cellobiohydrolase	

	Cellulase II	1.5	-	-	6-7.5	-	95	to 80	Exoglucanase Cellobiohydrolase	64
	CelA	302	-	-	5	4-7	85-90	to 95	Exoglucanase Cellobiohydrolase	65
	CelB	0.89	-	-	7	5-9	95	to 95	Exoglucanase Cellobiohydrolase	65
Thermatoga neapolitana	CelA	1219	0.25	1219	6	-	95	to 95	β-glucosidase Cellobiohydrolase	21
	CelB	1536	0.22	1536	6-6.6	-	106	to 106	β-glucosidase Cellobiohydrolase	21
Ergates faber	Cellulase A	30	20	-	4-4.7	-	37	to 55	None	87
	Cellulase B	56	28	-	5.2	-	37	to 45	None	87
	Cellulase C	157	6	-	5.2	-	37	to 60	None	87
Macrotermes mulleri	Cellulase IT	360	-	-	4.4	2.8-5.5	55	to 42	Exoglucanase	18
	Cellulase II	274	-	-	4.3-4.5	2.8-5.5	37	to 42	Exoglucanase	18
Panesthia cribrata	EG1	22.2	9.4	1850	-	-	-	-	None	88
	EG2	88.3	6.8	3440	-	-	-	-	None	88
Panesthia hilaris	Cellulase	150	-	-	5.5	4-7	-	-	None	89
Phaseolus vulgaris	Cellulase 4.5	17	-	-	5.7-6.2	6-9	35-43	to 30	None	90
	Cellulase 4.8	814	-	-	4.8-5.6	6.5-8	45-60	to 36	None	90

Table S5. Properties of enzymes with β-glucosidase activity. Table is organized by origin, fungal, then bacterial, then plant. All values listed are from enzymatic activities against *p*-nitrophenyl-β-D-glucopyranoside, PNPG. "-", not reported in the study. Rows of anaerobic fungi are highlighted.

Origin	Protein Name (Annotation)	Specific Activity (U/mg)	Kinetics Km (mM)	Kinetics Vmax (U/mg)	Optimal pH	pH Stability	Optimal Temp. (°C)	Temp. Stability (°C)	Other Activities	Ref.
Orpinomyces sp. strain C1A	BGL1	1.063	0.164	5.4	7	5-9	39	4-50	None	This Study
	BGL3	0.8	0.275	1.6	6	6-8	39	4-39	β-xylosidase	This study
	EG5	0.29	84	0.45	7	3-8	50	4-50	Endoglucanase Xylanase	This Study
	XYL11	0.22	0.216	0.28	5	3-9	50	4-60	Xylanase	This Study
	Bgxg1	73.4	0.000013	769	6	4-12	39	4-70	β-xylosidase β-galactosidase Xylanase	[91]
Acremonium persicinum	β-glucosidase	0.0183	0.3	-	5.5	-	-	-	Cellobiohydrolase	[45]
Aspergillus fumigatus	β-xylosidase	0.76	-	-	4.5	2-8	75	to 65	β-xylosidase	[92]
	β-Glucosidase	1.03	-	-	4.5	2-8	65	to 60	β-xylosidase	[92]
Aspergillus nidulans	B-Gluco-I	4.52	0.24	8	5	-	65	-	None	[1]
	B-Gluco-II	0.55	0.12	0.7	6	-	35	-	None	[1]
Aspergillus niger	β-xylosidase	0.15	-	-	6.7-7	-	42	to 46	β-xylosidase	[93]
	β-xylosidase 1	0.2	-	-	-	-	-	-	β-xylosidase Arabinosidase	[94]
	β-xylosidase 2	0.3	-	-	-	-	-	-	β-xylosidase Arabinosidase	[94]
Aureobasidium sp.	β-xylosidase	15.6	-	-	3.5	3.5-9	80	to 80	β-xylosidase	[95]
Candida peltata	β-glucosidase	108	2.3	221	5	3.5-6	50	25-45	Cellobiohydrolase	[46]
Chrysosporium lucknowense	EG60	0.12	-	-	4.5-6	5-7	60-70	60-75	Endoglucanase	[2]
	CBH1	0.02	-	-	4.5-6	5-7	60-70	60-75	Cellobiohydrolase	[2]
Fusarium oxysporum	Xylanase	0.9	-	-	7.4	5.8-8.2	50	to 50	Xylanase Endoglucanase Exoglucanase	[3]
Fusarium solani	Cellulase	4.65	-	-	5	5.7	65	to 65	Cellobiohydrolase Endoglucanase Exoglucanase	[4]
Humicola grisea	Exoglucanase 1	1.14	-	-	6-8	4-14	55-70	30-75	Cellobiohydrolase Endoglucanase Exoglucanase	[6]
	β-glucosidase 1	85.7	0.16	64	4.5	4-12	60-65	30-60	Cellobiohydrolase β-galactosidase β-xylosidase	[6]
	β-glucosidase 2	12.5	0.25	21	6-8	4-12	60-65	30-50	Cellobiohydrolase β-galactosidase β-xylosidase	[6]

	β-glucosidase 3	12.6	0.22	23	6-8	4-12	60-65	30-50	Cellobiohydrolase<br>β-galactosidase<br>β-xylosidase	6
	β-glucosidase 4	8.34	0.34	8.7	6-7	4-12	50-55	30-50	Cellobiohydrolase<br>β-galactosidase<br>β-xylosidase	6
	β-glucosidase 5	17.9	0.56	18	6	4-12	60	30-55	Cellobiohydrolase<br>β-galactosidase<br>β-xylosidase	6
	β-glucosidase 6	33.4	0.12	40	6-7	4-12	65-70	30-65	Cellobiohydrolase<br>β-galactosidase<br>β-xylosidase	6
	BGL I	26.1	0.34	25	6	6-11	55	to 50	Cellobiohydrolase<br>β-galactosidase	48
Neocallimastix frontalis	β-xylosidase	0.2	2.98	0.27	6.4	-	37	to 40	β-xylosidase<br>Arabinosidase	96
	β-glucosidase	9.0	0.031	-	-	-	-	-	β-galactosidase<br>Cellobiohydrolas	97
	β-glucosidase	15	0.55	-	6	5-8	50	to 40	None	98
Orpinomyces sp. strain PC-2	β-glucosidase	33.5	0.39	47.5	6.2	5-8	50	to 65	Cellobiohydrolase	99
Paecilomyces thermophila	β-glucosidase	97.2	0.27	780	6.2	5-8.5	75	65-70	Exoglucanase	51
Penicillium funiculosum	Cellulase	-	0.77	-	5	-	50	to 60	Cellobiohydrolase<br>Exoglucanase	14
Phanerochaete chrysosporium	β-glucosidase	-	0.096	-	4-5.2	4-8	25	to 45	None	53
Pichia pastoris X-33	EXG1	37.5	-	-	-	-	-	-	β-xylosidase<br>Exoglucanase	15
Piromyces communis	β-glucosidase	20	0.52	-	6	5-8	50	to 40	None	98
Piromyces sp. E2	β-glucosidase	0.25	1.0	66.5	5.7-6.3	-	47	to 37	Cellobiohydrolase	100
Sphaeromonas communis	β-glucosidase	46	0.51	-	6	5-8	50	to 40	None	98
Sporotrichum thermophila	β-glucosidase I	89	0.29	-	5.4	4-6.5	65	to 65	None	55
	β-glucosidase A	0.595	0.5	-	5.6	-	50	to 40	None	101
	β-glucosidase B	1.295	0.18	-	6.3	-	50	to 40	None	101
Stachybotrys sp.	β-glucosidase p21	0.33	0.27	78	5	5-7	50	to 60	None	56
Thermoascus aurantiacus	β-glucosidase	232	0.52	6500	5	6-8	70	to 75	None	72
	β-glucosidase	0.3	0.11	-	4.5	4.4-5.2	80	70-90	Cellobiohydrolase	102
Trichoderma harzianum	BGL	0.35	-	-	-	-	28	-	None	19
	β-xylosidase	0.28	-	-	4-4.5	-	60	-	β-xylosidase<br>β-galactosidase<br>Arabinosidase	103
Trichoderma koningii	β-glucosidase	16	0.37	-	4-5	4-6	45	to 65	Cellobiohydrolase	57
	β-glucosidase II	14	0.85	-	4-5	4-6	45	to 65	Cellobiohydrolase	57
Trichoderma reesei	BGL2	23.9	2.22	40	6	6-11	45	to 45	Cellobiohydrolase<br>β-galactosidase	48

	BGLI	767.7	-	-	4	5-9	70	to 55	Cellobiohydrlase	23
	CBH I	5.5	-	-	-	-	-	-	Cellobiohydrolase<br>Endoglucanase	59
	CBH II	33	-	-	-	-	-	-	Cellobiohydrolase<br>Endoglucanase	59
	CBH III	0.00001	-	-	-	-	-	-	Cellobiohydrolase<br>Xylanase<br>Mannanase<br>β-xylosidase<br>Exoglucanase	22
	EG I	0.00005	-	-	-	-	-	-	Cellobiohydrolase<br>Xylanase<br>Mannanase<br>β-xylosidase<br>Exoglucanase	22
	XYL 9	0.00001	-	-	-	-	-	-	Xylanase<br>Mannanase<br>β-xylosidase	22
	XYL 5.5	0.000001	-	-	-	-	-	-	Xylanase<br>Mannanase<br>β-xylosidase	22
Trichoderma viride T100-14	β-glucosidase	5.6	-	-	-	-	-	-	Cellobiohydrolase	25
Bacillus sp. KSM-S237	Cellulase	5.4	-	-	8.6-9	5-11	45	to 60	Endoglucanase<br>Cellobiohydrolase<br>Exoglucanase<br>Xylanase	76
Bacillus pumilus EB3	Cellulase	0.038	-	-	6	5-9	60	30-70	Exoglucanase<br>Endoglucanase	80
Bacillus sphaericus JS1	Cellulase	3.07	-	-	8	7-10.5	60	to 70	Endoglucanase	31
Cellulomonas fimi	CenA	1.6	-	-	-	-	-	-	Exoglucanase<br>Endoglucanase<br>Cellobiohydrolase	36
	Exg	0.9	-	-	6	5-9	-	60-75	Xylanase<br>Endoglucanase<br>Exoglucanase<br>Cellobiohydrolase	61
Clostridium stercorarium	β-glucosidase	31.6	0.8	-	5.5	5-7	65	to 75	Cellobiohydrolase	62
Clostridium thermocellum	β-glucosidase	127	-	-	6-6.5	5.5-7.5	60	to 68.5	Cellobiohydrolase	63
Thermotoga neapolitana	CelA	0.01	-	-	6	-	95	to 95	Endoglucanase<br>Cellobiohydrolase	21
	CelB	0.18	-	-	6-6.6	-	106	to 106	Endoglucanase<br>Cellobiohydrolase	21
Panesthia cribrata	GD1	9	10.6	-	-	-	-	-	Endoglucanase	88
	GD2	3	13.8	-	-	-	-	-	Endoglucanase	88

Table S6. Properties of enzymes with β-xylosidase activity. Table is organized by origin, fungal, then bacterial, then plant. All values listed are from enzymatic activities against *p*-nitrophenyl-β-D-xylopyranoside, PNPX. "-", not reported in the study. Rows of anaerobic fungi are highlighted.

Origin	Protein Name (Annotation)	Specific Activity (U/mg)	Kinetics Km (mM)	Kinetics Vmax (U/mg)	Optimal pH	pH Stability	Optimal Temp. (°C)	Temp. Stability (°C)	Other Activities	Ref.
Orpinomyces sp. strain C1A	BGL3	0.06	50.6	0.068	6	6-8	39	4-39	β-xylosidase	This study
	Bgxg1	11.5	0.00485	127	6	4-12	39	4-70	β-glucosidase<br>β-galactosidase<br>Xylanase	91
Acremonium persicinum	β-glucosidase	0.002	-	-	5.5	-	-	-	β-glucosidase	45
Aspergillus awamori	β-xylosidase	34.1	0.003	476	6.5	-	70	to 70	Xylanase	104
Aspergillus carnonarious	β-xylosidase	3.29	0.198	3.64	4	3.5-6.5	60	to 50	Arabinosidase	105
Aspergillus fumigatus	β-xylosidase	27.5	2	-	4.5	2-8	75	to 65	β-glucosidase	92
	β-Glucosidase	1.03	-	-	4.5	2-8	65	to 60	β-glucosidase	92
Aspergillus nidulans	β-xylosidase	1.2	1.1	25.6	5	4-6	50	to 45	None	106
Aspergillus niger	β-xylosidase	5.2	0.22	-	6.7-7	-	42	to 46	β-glucosidase	93
	β-xylosidase 1	60.2	-	-	-	-	-	-	β-glucosidase<br>Arabinosidase	94
	β-xylosidase 2	60.9	-	-	-	-	-	-	β-glucosidase<br>Arabinosidase	94
Aspergillus phoenicis	β-xylosidase	821	2.36	-	4-4.5	4-6	75	to 60	None	107
Aspergillus pulverulentus	β-xylI	32	-	-	2.5-3.5	1.5-6.5	60	to 50	None	108
	β-xylII	12	-	-	4-5	1.5-6.5	60	to 50	None	108
Aureobasidium sp.	β-xylosidase	626	2	940	3.5	3.5-9	80	to 80	β-glucosidase	95
Aureobasidium pullulans	β-xylosidase	7.3	-	-	4.5	2-9.5	80	to 70	None	109
Chaetomonium cellulolyticum	Xylanase 3	0.03	-	-	6-7	-	50	-	Xylanase<br>Arabinosidase<br>Endoglucanase	47
Fusarium oxysporum	Xylanase	0.5	-	-	7.4	5.8-8.2	50	to 50	Xylanase<br>Endoglucanase<br>Exoglucanase	3
Fusarium proliferatum	β-xylosidase	53	0.77	-	4.5	-	60	-	None	110
Fusarium vercillioides	β-xylosidase	57	0.85	-	4.5	-	65	-	None	111
Humicola grisea	β-glucosidase 1	4.38	-	-	4.5	4-12	60-65	30-60	β-glucosidase<br>β-galactosidase<br>Cellobiohydrolase	6
	β-glucosidase 2	0.07	-	-	6-8	4-12	60-65	30-50	β-glucosidase<br>β-galactosidase<br>Cellobiohydrolase	6
	β-glucosidase 3	0.08	-	-	6-8	4-12	60-65	30-50	β-glucosidase<br>β-galactosidase<br>Cellobiohydrolase	6

	β-glucosidase 4	0.21	-	-	6-7	4-12	50-55	30-50	β-glucosidase<br>β-galactosidase<br>Cellobiohydrolase	6
	β-glucosidase 5	0.36	-	-	6	4-12	60	30-55	β-glucosidase<br>β-galactosidase<br>Cellobiohydrolase	6
	β-glucosidase 6	0.17	-	-	6-7	4-12	65-70	30-65	β-glucosidase<br>β-galactosidase<br>Cellobiohydrolase	6
	β-xylosidase	19.6	1.37	13	6.5	4-9	55	to 50	None	112
Neocallimastix frontalis	β-xylosidase	4.3	0.33	-	6.5	-	35	-	Xylanase	113
	β-xylosidase	0.9	2.98	0.27	6.4	-	37	to 40	β-glucosidase<br>Arabinosidase	96
	β-xylosidase	16	0.34	-	6.5	5-8	35	to 40	None	98
Neocallimastix patriciarum	β-xylosidase I	30.4	0.59	38	6	5-8	50	45-60	Arabinosidase	114
	β-xylosidase II	8.7	0.13	8.9	6	5-7	40	25-45	None	114
Neurospora crassa	β-xylosidase	0.26	0.047	-	4.5-5	-	55	-	Cellobiohydrolase	115
Penicillium wortmanni	Xylosidase 1	22	4.2	42.6	3-4.5	-	55-65	-	None	116
	Xylosidase 2	40	2.3	73.2	3-4.5	-	55-65	-	None	116
	Xylosidase 3	61.5	1.0	88.2	3-4.5	-	55-65	-	None	116
	Xylosidase 4	30.5	3.3	106.6	3-4.5	-	55-65	-	None	116
Pichia pastoris X-33	EXG1	11.6	-	-	-	-	-	-	β-glucosidase<br>β-galactosidase	15
Piromyces communis	β-xylosidase	28	0.4	-	6.0	5-8	39	to 40	None	98
Sphaeromonas communis	β-xylosidase	27	0.36	-	6.5	5-8	39	to 40	None	98
Talaomyces emersonii	Xyl I	3.0	0.13	-	2.5	-	60	-	None	117
	Xyl II	92.9	32.9	-	4.2	-	78	-	None	117
	Xyl III	0.2	1.4	-	3.5	-	67	-	None	117
Trichoderma harzianum	β-xylosidase	3.42	0.103	-	4-4.5	-	60	-	β-glucosidase<br>β-galactosidase<br>Arabinosidase	103
Trichoderma reesei	CBHII	0.00001	-	-	-	-	-	-	β-glucosidase<br>Mannanase<br>Xylanase<br>Cellobiohydrolase<br>Exoglucanase	22
	EGI	0.00001	-	-	-	-	-	-	β-glucosidase<br>Mannanase<br>Xylanase<br>Cellobiohydrolase<br>Exoglucanase	22
	XYL 9	0.00002	-	-	-	-	-	-	β-glucosidase<br>Mannanase<br>Xylanase	22

	XYL 5.5	0.00005	-	-	-	-	-	-	β-glucosidase<br>Mannanase<br>Xylanase	22
	β-xylosidase	470	0.08	-	4	3-7	60	to 55	Arabinosidase	118
Trichoderma viride	β-xylosidase	10.8	-	-	3.5	4-5	55	to 65	None	119
Bacillus halodurans C-125	BH1068	-	30.1	-	6.5	5-7	47	to 45	None	120
Bacillus stearothermophilus	β-xylosidase	34.2	0.0012	-	6	6-8	70	to 60	None	121
Bacillus thermantarcticus	β-xylosidase	160	0.5	-	6.0	-	70	to 60	None	122
Butyrivibrio fibrisolvens	xylB	8.9	-	-	-	-	-	-	Arabinosidase	123
Caulobacter crescentus	CcXynB2	215	9.3	402	6	4.5-7.5	55	to 50	None	124
Clostridium stercorarium	β-glucosidase	5.7	-	-	5.5	5-7	65	to 75	β-glucosidase<br>Cellobiohydrolase<br>Exoglucanase	62
	xylA	3.5	2.5	5.9	7	5-10	65	-	Arabinosidase	125
Geobacillus stearothermophilus	β-xylosidase	133	2.38	147	6.5	5.5-7	70	50-70	None	126,127
Ruminococcus flavefaciens	Exo A	0.01	-	-	5	4.5-5.5	-	-	Cellobiohydrolase<br>Exoglucanase	43
Thermoanaerobacterium saccharolyticum	β-xylosidase	53.8	28	276	6	6-6.5	65	to 67	None	128
Thermoanaerobacter sp.	β-xylosidase	66	0.038	183	5.9	5-8.5	93	to 86	Arabinosidase	129
Hordeum vulgare	β-xylosidase	6.06	-	-	-	-	-	-	Arabinosidase	130

Table S7. Properties of enzymes with xylanase activity. Table is organized by origin, fungal, then bacterial, then plant. All values listed are from enzymatic activities against xylan. "-", not reported in the study. Rows of anaerobic fungi are highlighted.

Origin	Protein Name (Annotation)	Specific Activity (U/mg)	Kinetics Km (mg/mL)	Kinetics Vmax (U/mg)	Optimal pH	pH Stability	Optimal Temp. (°C)	Temp. Stability (°C)	Other Activities	Ref.
Orpinomyces sp. strain C1A	XYL11	73.5	0.024	370	5	3-9	50	4-60	β-glucosidase	This Study
	EG5	9.37	0.035	36.5	7	3-8	50	4-50	Endoglucanase<br>β-glucosidase	This Study
	Bgxg1	10.8	0.038	25.6	6	4-12	39	4-70	β-glucosidase<br>β-xylosidase<br>β-galactosidase	91
Acrophialophora nainiana	Xyn III	31.25	4.37	0.24	6.5	-	50	to 55	None	131
	Xyn II	33.2	40.9	-	7	-	55	to 55	None	132
	Xyn I	13.6	0.731	-	6	-	50-60	to 50	None	133
Aspergillus awamori	β-xylosidase	1.7	-	-	6.5	-	70	to 70	β-xylosidase	104
Aspergillus niger	Xylanase I	18.9	-	-	6-6.5	-	65-80	to 80	Exoglucanase	93
	Xylanase IA	35.2	-	-	5.5-6	-	65-80	to 80	Exoglucanase	93
	Xylanase II	24.5	-	-	4-4.5	-	65-80	to 80	None	93
	Xylanase IIA	48.0	-	-	4	-	65-80	to 80	None	93
Chaetomonium cellulolyticum	Xylanase 1	22	-	-	6-7	-	50	-	Endoglucanase<br>Exoglucanase	47
	Xylanase 2	18	-	-	6-7	-	50	-	Endoglucanase<br>Exoglucanase	47
	Xylanase 3	11	-	-	6-7	-	50	-	Endoglucanase<br>Arabinosidase	47
Chrysosporium lucknowense	EG28	0.2	-	-	4.5-6	5-7	60-70	60-75	Endoglucanase<br>Exoglucanase	2
	EG44	0.07	-	-	4.5-6	5-7	60-70	60-75	Endoglucanase<br>Exoglucanase	2
	EG47	0.08	-	-	4.5-6	5-8.5	60-70	60-75	Endoglucanase<br>Exoglucanase	2
	EG51	0.18	-	-	4.5-6	5-7	60-70	60-75	Endoglucanase<br>Exoglucanase	2
	CBHII	1.4	-	-	4.5-6	5-7	60-70	60-75	Cellobiohydrolase<br>Exoglucanase	2
Fusarium oxysporum	Xylanase	5.2	3.8	-	7.4	5.8-8.2	50	to 50	Endoglucanase<br>Exoglucanase	3
Gloephyllum trabeum	Cel5A	0.77	-	-	-	-	-	-	Endoglucanase<br>Exoglucanase	5
	Xyn10A	390	-	-	-	-	-	-	Endoglucanase<br>Exoglucanase	5

	Cel12A	0.88	-	-	-	-	-	-	Endoglucanase<br>Exoglucanase	[5]
Humicola grisea	X1	-	7.233		5		50	to 50	None	[134]
	X2	10.23	10.87	-	4.5-6.5	-	55-60	to 60	None	[134]
	Xylanase	169.4	3.3	229	5.5	-	70	to 50	None	[135]
Neocallimastix frontalis	Xylanase	0.36	1.13	-	6	3-8	50	to 40	None	[136]
Orpinomyces joyonii	Cellulase	5.2	-	-	5.5	5-7.5	40	to 50	Exoglucanase<br>Endoglucanase<br>Cellobiohydrolase	[49]
Paecilomyces thermophila	Xylanase	2063	2	2344	7	6-10	75-80	to 75	None	[137]
Penicillium brasilianum	XYL	279	-	-	-	-	-	-	None	[13]
Penicillium citrinum	Xylanase	360.7	-	-	8.5	4-10	50	0-60	None	[138]
Piromyces communis	Xylanase	0.37	1	-	5.5	-	50	-	None	[136]
Sphaeromonas communis	Xylanase	0.43	0.98	-	6	-	50	-	None	[136]
Trichoderma harzianum	XYL2	-	13.66	-	5	3.5-6.5	45	to 50	None	[139]
	Xylanase	2400	-	-	-	-	-	-	None	[140]
Trichoderma reesei	EG I	0.55	-	-	-	-	-	-	Endoglucanase<br>Cellobiohydrolase<br>β-glucosidase	[60]
	EG II	0.003	-	-	-	-	-	-	Endoglucanase<br>Cellobiohydrolase<br>β-glucosidase	[60]
	CBH I	0.0003	-	-	-	-	-	-	Endoglucanase<br>Cellobiohydrolase<br>β-glucosidase	[60]
	CBH II	0.002	-	-	-	-	-	-	Endoglucanase<br>Cellobiohydrolase<br>β-glucosidase	[60]
	CBH III	0.0002	-	-	-	-	-	-	Endoglucanase<br>β-glucosidase<br>β-xylosidase	[22]
	EG I	0.29	-	-	-	-	-	-	Endoglucanase<br>β-glucosidase<br>β-xylosidase	[22]
	XYL 9	11.33	-	-	-	-	-	-	Endoglucanase<br>β-glucosidase<br>β-xylosidase	[22]
	XYL 5.5	3.855	-	-	-	-	-	-	Endoglucanase<br>β-glucosidase<br>β-xylosidase	[22]
	EG I	46.2	-	-	4	2-8	60	to 45	Endoglucanase<br>Exoglucanase	[23]

									Cellobiohydrolase	
Anaerocellum thermophilum	CelA	0.372	-	-	5-6	-	85-95	-	Endoglucanase<br>Exoglucanase	[26]
Bacillus amyoliquefaciens	Cellulase	22.5	-	-	7	4-9	50	40-80	Endoglucanase<br>Exoglucanase<br>Cellobiohydrolase<br>β-glucosidase	[27]
Bacillus circulans	XylA	2039.5	4	2667	6-6.5	4-8	75-80	to 65	None	[141]
	XylB	6423	25	200000	6-6.5	4-8	65-70	to 65	None	[141]
Bacillus licheniformis	Xylanase	28.7	3.33	111	6-7.5	5-8	60	to 60	None	[142]
Bacillus stearothermophilus	Xylanase	122	3.8	-	7	5-11	60	to 60	None	[121]
Bacillus subtilis	Cellulase	980	-	-	6	6-7.5	50-60	to 50	Endoglucanase	[32]
Bacillus thermantarcticus	Xylanase	141	1.6	-	5.6	-	80	to 60	None	[122]
Caldibacillus cellulovorans	CMCase	4.56	-	-	6.5-7	-	80	to 70	Endoglucanase<br>Exoglucanase	[33]
Cellulomonas fimi	Exg	37	-	-	6	5-9	-	60-75	Endoglucanase<br>Cellobiohydrolase	[61]
Clostridium stercorarium	Avicelase II	0.340	-	-	5-6	4.5-7	75	65-80	Exoglucanase	[38]
Streptomyces lividans	Xylanase	364	0.78	850	6	5.5-7	60	0-37	None	[143]
Streptomyces rameus	Xylanase	4326	5.8	-	5.3	4.3-6.7	70	40-65	None	[144]
Streptomyces thermoviolaceus	STX-I	1460	-	-	7	5-9	70	to 50	None	[145]
	STX-II	1405	-	-	7	5-9	60	to 60	None	[145]
Thermatoga maritima	CelA	2.5	-	-	5	4-9	85-90	to 95	Endoglucanase<br>Exoglucanase<br>Cellobiohydrolase	[65]
	CelB	0.008	-	-	7	5-9	85-90	to 95	Endoglucanase<br>Exoglucanase<br>Cellobiohydrolase	[65]
Thermatoga neapolitana	CelB	37	-	-	6-6.6	-	106	to 106	Endoglucanase<br>Exoglucanase<br>Cellobiohydrolase	[21]

Supplemental Information References

1 Bagga, P. S., Sandhu, D. K. & Sharma, S. Purification and characterization of cellulolytic enzymes produced by *Aspergillus nidulans*. *J Appl Bacteriol* **68**, 61-68, doi:DOI 10.1111/j.1365-2672.1990.tb02549.x (1990).

2 Bukhtojarov, F. E. *et al.* Cellulase complex of the fungus *Chrysosporium lucknowense*: Isolation and characterization of endoglucanases and cellobiohydrolases. *Biochemistry* **69**, 542-551, doi:Doi 10.1023/B:Biry.0000029853.34093.13 (2004).

3 Christakopoulos, P., Kekos, D., Macris, B. J., Claeyssens, M. & Bhat, M. K. Purification and characterisation of a major xylanase with cellulase and transferase activities from *Fusarium oxysporum*. *Carbohyd Res* **289**, 91-104, doi:Doi 10.1016/0008-6215(96)00146-2 (1996).

4 Wood, T. M. Cellulase of *Fusarium solani*. *Biochem J* **121**, 353-362 (1971).

5 Cohen, R., Suzuki, M. R. & Hammel, K. E. Processive endoglucanase active in crystalline cellulose hydrolysis by the brown rot basidiomycete *Gloeophyllum trabeum*. *Appl Environ Microbiol* **71**, 2412-2417, doi:10.1128/Aem.71.5.2412-2417.2005 (2005).

6 Takashima, S., Nakamura, A., Masaki, H. & Uozumi, T. Purification and characterization of cellulases from *Humicola grisea*. *Biosci Biotech Bioch* **60**, 77-82 (1996).

7 Wang, H. C., Chen, Y. C., Huang, C. T. & Hseu, R. S. Cloning and characterization of a thermostable and pH-stable cellobiohydrolase from *Neocallimastix patriciarum* J11. *Protein Expres Purif* **90**, 153-159, doi:10.1016/j.pep.2013.06.004 (2013).

8 Xue, G. P., Orpin, C. G., Gobius, K. S., Aylward, J. H. & Simpson, G. D. Cloning and expression of multiple cellulase cDNAs from the anaerobic rumen fungus *Neocallimastix patriciarum* in *Escherichia coli*. *J Gen Microbiol* **138**, 1413-1420 (1992).

9 Xue, G. P., Gobius, K. S. & Orpin, C. G. A novel polysaccharide hydrolase Cdna (Celd) from *Neocallimastix patriciarum* encoding 3 multifunctional catalytic domains with high endoglucanase, cellobiohydrolase and xylanase activities. *J Gen Microbiol* **138**, 2397-2403 (1992).

10 Liu, J. H. *et al.* An endoglucanase from the anaerobic fungus *Orpinomyces joyonii:* characterization of the gene and its product. *Can J Microbiol* **43**, 477-485 (1997).

11 Li, X. L., Chen, H. Z. & Ljungdahl, L. G. Two cellulases, CelA and CelC, from the polycentric anaerobic fungus *Orpinomyces* strain PC-2 contain N-terminal docking domains for a cellulase-hemicellulase complex. *Appl Environ Microbiol* **63**, 4721-4728 (1997).

12 Chen, Y. C., Chen, W. T., Liu, J. C., Tsai, L. C. & Cheng, H. L. A highly active beta-glucanase from a new strain of rumen fungus *Orpinomyces* sp.Y102 exhibits cellobiohydrolase and cellotriohydrolase activities. *Bioresource Technol* **170**, 513-521, doi:10.1016/j.biortech.2014.08.016 (2014).

13 Jorgensen, H., Eriksson, T., Borjesson, J., Tjerneld, F. & Olsson, L. Purification and characterization of five cellulases and one xylanase from *Penicillium brasilianum* IBT 20888. *Enzyme Microb Tech* **32**, 851-861, doi:10.1016/S0141-0229(03)00056-5 (2003).

14 Wood, T. M. & Mccrae, S. I. Purification and some properties of a (1-]4)-beta-D-glucan glucohydrolase associated with the cellulase from the fungus *Penicillium funiculosum*. *Carbohyd Res* **110**, 291-303, doi:Doi 10.1016/0008-6215(82)84011-1 (1982).

15 Xu, Z., Shih, M. C. & Poulton, J. E. An extracellular exo-beta-(1,3)-glucanase from *Pichia pastoris:* purification, characterization, molecular cloning, and functional expression. *Protein Expr Purif* **47**, 118-127, doi:10.1016/j.pep.2005.11.025 (2006).

16 Chu, C. Y., Tseng, C. W., Yueh, P. Y., Duan, C. H. & Liu, J. R. Molecular cloning and characterization of a beta-glucanase from *Piromyces rhizinflatus*. *J Biosci Bioeng* **111**, 541-546, doi:10.1016/j.jbiosc.2011.01.009 (2011).
17 Harhangi, H. R. *et al.* Cel6A, a major exoglucanase from the cellulosome of the anaerobic fungi *Piromyces* sp. E2 and *Piromyces equi*. *Biochim Biophys Acta* **1628**, 30-39 (2003).
18 Rouland, C., Civas, A., Renoux, J. & Petek, F. Purification and properties of cellulases from the termite *Macrotermes mulleri* (Termitidae, Macrotermitinae) and its symbiotic fungus *Termitomyces* sp. *Comp Biochem Phys B* **91**, 449-458, doi:Doi 10.1016/0305-0491(88)90004-1 (1988).
19 Ahmed, S., Bashir, A., Saleem, H., Saadia, M. & Jamil, A. Production and purification of cellulose-degrading enzymes from a filamentous fungus *Trichoderma harzianum*. *Pak J Bot* **41**, 1411-1419 (2009).
20 Thrane, C., Tronsmo, A. & Jensen, D. F. Endo-1,3-beta-glucanase and cellulase from *Trichoderma harzianum*: purification and partial characterization, induction of and biological activity against plant pathogenic *Pythium* spp. *Eur J Plant Pathol* **103**, 331-344, doi:Doi 10.1023/A:1008679319544 (1997).
21 Bok, J. D., Yernool, D. A. & Eveleigh, D. E. Purification, characterization, and molecular analysis of thermostable cellulases CelA and CelB from *Thermotoga neapolitana*. *Appl Environ Microbiol* **64**, 4774-4781 (1998).
22 Bailey, M. J., Siikaaho, M., Valkeajarvi, A. & Penttila, M. E. Hydrolytic properties of 2 cellulases of *Trichoderma reesei* expressed in yeast. *Biotechnol Appl Bioc* **17**, 65-76 (1993).
23 Takashima, S. *et al.* Overproduction of recombinant *Trichoderma reesei* cellulases by *Aspergillus oryzae* and their enzymatic properties. *J Biotechnol* **65**, 163-171, doi:Doi 10.1016/S0168-1656(98)00084-4 (1998).
24 Tomme, P. *et al.* Studies of the cellulolytic system of *Trichoderma reesei* qm-9414 - analysis of domain function in 2 cellobiohydrolases by limited proteolysis. *Eur J Biochem* **170**, 575-581, doi:DOI 10.1111/j.1432-1033.1988.tb13736.x (1988).
25 Zhou, J. *et al.* Identification and purification of the main components of cellulases from a mutant strain of *Trichoderma viride* T 100-14. *Bioresource Technol* **99**, 6826-6833, doi:10.1016/j.biortech.2008.01.077 (2008).
26 Zverlov, V., Mahr, S., Riedel, K. & Bronnenmeier, K. Properties and gene structure of a bifunctional cellulolytic enzyme (CelA) from the extreme thermophile '*Anaerocellum thermophilum*' with separate glycosyl hydrolase family 9 and 48 catalytic domains. *Microbiol* **144**, 457-465 (1998).
27 Lee, Y. J. *et al.* Purification and characterization of cellulase produced by *Bacillus amyoliquefaciens* DL-3 utilizing rice hull. *Bioresource Technol* **99**, 378-386, doi:10.1016/j.biortech.2006.12.013 (2008).
28 Mawadza, C., Hatti-Kaul, R., Zvauya, R. & Mattiasson, B. Purification and characterization of cellulases produced by two *Bacillus* strains. *J Biotechnol* **83**, 177-187, doi:Doi 10.1016/S0168-1656(00)00305-9 (2000).
29 Ito, S. Alkaline cellulases from alkaliphilic *Bacillus*: Enzymatic properties, genetics, and application to detergents. *Extremophiles* **1**, 61-66, doi:DOI 10.1007/s007920050015 (1997).

30 Han, S. J., Yoo, Y. J. & Kang, H. S. Characterization of a bifunctional cellulase and its structural gene - the Cel gene of *Bacillus* sp D04 has exoglucanase and endoglucanase activity. *J Biol Chem* **270**, 26012-26019 (1995).

31 Singh, J., Batra, N. & Sobti, R. C. Purification and characterisation of alkaline cellulase produced by a novel isolate, *Bacillus sphaericus* JS1. *J Ind Microbiol Biot* **31**, 51-56, doi:10.1007/s10295-004-0114-0 (2004).

32 Yin, L. J., Lin, H. H. & Xiao, Z. R. Purification and characterization of a cellulase from *Bacillus subtilis* yj1. *J Mar Sci Tech* **18**, 466-471 (2010).

33 Huang, X. P. & Monk, C. Purification and characterization of a cellulase (CMCase) from a newly isolated thermophilic aerobic bacterium *Caldibacillus cellulovorans* gen. nov., sp nov. *World J Microb Biot* **20**, 85-92, doi:Doi 10.1023/B:Wibi.0000013316.12730.E7 (2004).

34 MacLeod, A. M., Lindhorst, T., Withers, S. G. & Warren, R. A. The acid/base catalyst in the exoglucanase/xylanase from *Cellulomonas fimi* is glutamic acid 127: evidence from detailed kinetic studies of mutants. *Biochemistry* **33**, 6371-6376 (1994).

35 Nikolova, P. V., Creagh, A. L., Duff, S. J. & Haynes, C. A. Thermostability and irreversible activity loss of exoglucanase/xylanase Cex from *Cellulomonas fimi*. *Biochemistry* **36**, 1381-1388, doi:10.1021/bi962367f (1997).

36 Tomme, P., Kwan, E., Gilkes, N. R., Kilburn, D. G. & Warren, R. A. Characterization of CenC, an enzyme from *Cellulomonas fimi* with both endo- and exoglucanase activities. *J Bacteriol* **178**, 4216-4223 (1996).

37 Reverbel-Leroy, C., Pages, S., Belaich, A., Belaich, J. P. & Tardif, C. The processive endocellulase CelF, a major component of the *Clostridium cellulolyticum* cellulosome: purification and characterization of the recombinant form. *J Bacteriol* **179**, 46-52 (1997).

38 Bronnenmeier, K., Rucknagel, K. P. & Staudenbauer, W. L. Purification and properties of a novel type of exo-1,4-beta-glucanase (avicelase II) from the cellulolytic thermophile *Clostridium stercorarium*. *Eur J Biochem* **200**, 379-385 (1991).

39 Arai, T. *et al.* Characterization of a cellulase containing a family 30 carbohydrate-binding module (CBM) derived from *Clostridium thermocellum* CelJ: Importance of the CBM to cellulose hydrolysis. *J Bacteriol* **185**, 504-512, doi:10.1128/Jb.185.2.504-512.2003 (2003).

40 Kruus, K., Wang, W. K., Ching, J. & Wu, J. H. Exoglucanase activities of the recombinant *Clostridium thermocellum* CelS, a major cellulosome component. *J Bacteriol* **177**, 1641-1644 (1995).

41 Ng, T. K. & Zeikus, J. G. Purification and characterization of an endoglucanase (1,4-beta-D-glucan glucanohydrolase) from *Clostridium thermocellum*. *Biochem J* **199**, 341-350 (1981).

42 Bakare, M. K., Adewale, I. O., Ajayi, A. & Shonukan, O. O. Purification and characterization of cellulase from the wild-type and two improved mutants of *Pseudomonas fluorescens*. *Afr J Biotechnol* **4**, 898-904 (2005).

43 Gardner, R. M., Doerner, K. C. & White, B. A. Purification and characterization of an exo-beta-1,4-glucanase from *Ruminococcus flavefaciens* FD-1. *J Bacteriol* **169**, 4581-4588 (1987).

44 MacKenzie, C. R., Bilous, D. & Johnson, K. G. Purification and characterization of an exoglucanase from *Streptomyces flavogriseus*. *Can J Microbiol* **30**, 1171-1178 (1984).

45 Pitson, S. M., Seviour, R. J. & McDougall, B. M. Purification and characterization of an extracellular beta-glucosidase from the filamentous fungus *Acremonium persicinum* and its probable role in beta-glucan degradation. *Enzyme Microb Tech* **21**, 182-190, doi:Doi 10.1016/S0141-0229(96)00263-3 (1997).

46 Saha, B. C. & Bothast, R. J. Production, purification, and characterization of a highly glucose-tolerant novel beta-glucosidase from *Candida peltata*. *Appl Environ Microbiol* **62**, 3165-3170 (1996).

47 Baraznenok, V. A., Becker, E. G., Ankudimova, N. V. & Okunev, N. N. Characterization of neutral xylanases from *Chaetomium cellulolyticum* and their biobleaching effect on eucalyptus pulp. *Enzyme Microb. Technol.* **25**, 651-659, doi:Doi 10.1016/S0141-0229(99)00091-5 (1999).

48 Takashima, S., Nakamura, A., Hidaka, M., Masaki, H. & Uozumi, T. Molecular cloning and expression of the novel fungal beta-glucosidase genes from *Humicola grisea* and *Trichoderma reesei*. *J Biochem* **125**, 728-736 (1999).

49 Ye, X. Y., Ng, T. B. & Cheng, K. J. Purification and characterization of a cellulase from the ruminal fungus *Orpinomyces joyonii* cloned in *Escherichia coli*. *Int J Biochem Cell B* **33**, 87-94, doi:Doi 10.1016/S1357-2725(00)00068-6 (2001).

50 Chen, H. Z., Li, X. L., Blum, D. L. & Ljungdahl, L. G. Two genes of the anaerobic fungus *Orpinomyces* sp. strain PC-2 encoding cellulases with endoglucanase activities may have arisen by gene duplication. *FEMS Microbiol Lett* **159**, 63-68, doi:DOI 10.1111/j.1574-6968.1998.tb12842.x (1998).

51 Yang, S., Jiang, Z., Yan, Q. & Zhu, H. Characterization of a thermostable extracellular beta-glucosidase with activities of exoglucanase and transglycosylation from *Paecilomyces thermophila*. *J Agric Food Chem* **56**, 602-608, doi:10.1021/jf072279+ (2008).

52 Wood, T. M., Mccrae, S. I. & Macfarlane, C. C. The Isolation, purification and properties of the cellobiohydrolase component of *Penicillium funiculosum* cellulase. *Biochem J* **189**, 51-65 (1980).

53 Lymar, E. S., Li, B. & Renganathan, V. Purification and characterization of a cellulose-binding beta-glucosidase from cellulose-degrading cultures of *Phanerochaete chrysosporium*. *Appl Environ Microbiol* **61**, 2976-2980 (1995).

54 Eberhardt, R. Y., Gilbert, H. J. & Hazlewood, G. P. Primary sequence and enzymic properties of two modular endoglucanases, Cel5A and Cel45A, from the anaerobic fungus *Piromyces equi*. *Microbiol* **146**, 1999-2008 (2000).

55 Bhat, K. M., Gaikwad, J. S. & Maheshwari, R. Purification and characterization of an extracellular beta-glucosidase from the thermophilic fungus *Sporotrichum thermophile* and its influence on cellulase activity. *J Gen Microbiol* **139**, 2825-2832 (1993).

56 Amouri, B. & Gargouri, A. Characterization of a novel beta-glucosidase from a *Stachybotrys* strain. *Biochem Eng J* **32**, 191-197, doi:10.1016/j.bej.2006.09.022 (2006).

57 Wood, T. M. & Mccrae, S. I. Purification and some properties of the extracellular beta-D-glucosidase of the celluloytic fungus *Trichoderma koningii*. *J Gen Microbiol* **128**, 2973-2982 (1982).

58 Irwin, D. C., Spezio, M., Walker, L. P. & Wilson, D. B. Activity studies of 8 purified cellulases - specificity, synergism, and binding domain effects. *Biotechnol Bioeng* **42**, 1002-1013, doi:DOI 10.1002/bit.260420811 (1993).

59 Woodward, J., Brown, J. P., Evans, B. R. & Affholter, K. A. Papain digestion of crude *Trichoderma reesei* cellulase - purification and properties of cellobiohydrolase-I and cellobiohydrolase-II core proteins. *Biotechnol Appl Bioc* **19**, 141-153 (1994).
60 Suurnakki, A. *et al. Trichoderma reesei* cellulases and their core domains in the hydrolysis and modification of chemical pulp. *Cellulose* **7**, 189-209, doi:Doi 10.1023/A:1009280109519 (2000).
61 Curry, C., Gilkes, N., O'Neill, G., Miller, R. C. & Skipper, N. Expression and secretion of a *Cellulomonas fimi* exoglucanase in *Saccharomyces cerevisiae*. *Appl Environ Microbiol* **54**, 476-484 (1988).
62 Bronnenmeier, K. & Staudenbauer, W. L. Purification and properties of an extracellular beta-glucosidase from the cellulolytic thermophile *Clostridium stercorarium*. *Appl Microbiol Biot* **28**, 380-386 (1988).
63 Ait, N., Creuzet, N. & Cattaneo, J. Properties of beta-glucosidase purified from *Clostridium thermocellum*. *J Gen Microbiol* **128**, 569-577 (1982).
64 Bronnenmeier, K., Kern, A., Liebl, W. & Staudenbauer, W. L. Purification of *Thermotoga maritima* enzymes for the degradation of cellulosic materials. *Appl Environ Microbiol* **61**, 1399-1407 (1995).
65 Liebl, W. *et al.* Analysis of a *Thermotoga maritima* DNA fragment encoding two similar thermostable cellulases, CelA and CelB, and characterization of the recombinant enzymes. *Microbiol-SGM* **142**, 2533-2542 (1996).
66 Pauly, M. *et al.* A xyloglucan-specific endo-beta-1,4-glucanase from *Aspergillus aculeatus:* expression cloning in yeast, purification and characterization of the recombinant enzyme. *Glycobiology* **9**, 93-100, doi:DOI 10.1093/glycob/9.1.93 (1999).
67 Akiba, S., Kimura, Y., Yamamoto, K. & Kumagai, H. Purification and characterization of a protease-resistant cellulase from *Aspergillus niger*. *J Ferment Bioeng* **79**, 125-130, doi:Doi 10.1016/0922-338x(95)94078-6 (1995).
68 Hurst, P. L., Nielsen, J., Sullivan, P. A. & Shepherd, M. G. Purification and properties of a cellulase from *Aspergillus niger*. *Biochem J* **165**, 33-41 (1977).
69 Okada, G. Purification and properties of a cellulase from *Aspergillus niger*. *Agr Biol Chem* **49**, 1257-1265 (1985).
70 Thongekkaew, J., Ikeda, H., Masaki, K. & Iefuji, H. An acidic and thermostable carboxymethyl cellulase from the yeast *Cryptococcus* sp S-2: Purification, characterization and improvement of its recombinant enzyme production by high cell-density fermentation of *Pichia pastoris*. *Protein Expres Purif* **60**, 140-146, doi:10.1016/j.pep.2008.03.021 (2008).
71 Jin, X., Meng, N. & Xia, L. M. Expression of an endo-beta-1,4-glucanase gene from *Orpinomyces* PC-2 in *Pichia pastoris*. *Int J Mol Sci* **12**, 3366-3380, doi:10.3390/ijms12053366 (2011).
72 Tong, C. C., Cole, A. L. & Shepherd, M. G. Purification and properties of the cellulases from the thermophilic fungus *Thermoascus aurantiacus*. *Biochem J* **191**, 83-94 (1980).
73 Liu, J. & Xia, W. S. Purification and characterization of a bifunctional enzyme with chitosanase and cellulase activity from commercial cellulase. *Biochem Eng J* **30**, 82-87, doi:10.1016/j.bej.2006.02.005 (2006).
74 Muhammad, A., Iqbal, M., Mahmood, T. & Qadir, J. Calculation and measurement of kinetic parameters of Pakistan Research Reactor-1 (PARR-1). *Ann Nucl Energy* **38**, 44-48, doi:10.1016/j.anucene.2010.08.019 (2011).

75 Fukumori, F., Kudo, T. & Horikoshi, K. Purification andproperties of a cellulase from alkalophilic *Bacillus* sp no-1139. *J Gen Microbiol* **131**, 3339-3345 (1985).
76 Hakamada, Y. *et al.* Thermostable alkaline cellulase from an alkaliphilic isolate, *Bacillus* sp. KSM-S237. *Extremophiles* **1**, 151-156, doi:DOI 10.1007/s007920050028 (1997).
77 Kim, J. Y., Hur, S. H. & Hong, J. H. Purification and characterization of an alkaline cellulase from a newly isolated alkalophilic *Bacillus* sp HSH-810. *Biotechnol Lett* **27**, 313-316, doi:10.1007/s10529-005-0685-5 (2005).
78 Okoshi, H. *et al.* Purification and characterization of multiple carboxymethyl cellulases from *Bacillus* sp ksm-522. *Agr Biol Chem* **54**, 83-89 (1990).
79 Vijayaraghavan, P. & Vincent, S. G. P. Purification and characterization of carboxymethyl cellulase from *Bacillus* sp isolated from a paddy field. *Pol J Microbiol* **61**, 51-55 (2012).
80 Ariffin, H., Abdullah, N., Umi Kalsom, M. S., Shirai, Y. & Hassan, M. A. Production and characterization of cellulase by *Bacillus pumilus* EB3. *Int J Res Eng Technol* **3**, 47-53 (2006).
81 Li, W., Zhang, W. W., Yang, M. M. & Chen, Y. L. Cloning of the thermostable cellulase gene from newly isolated *Bacillus subtilis* and its expression in *Escherichia coli*. *Mol Biotechnol* **40**, 195-201, doi:10.1007/s12033-008-9079-y (2008).
82 Beguin, P. & Eisen, H. Purification and partial characterization of 3 extracellular cellulases from *Cellulomonas* sp. *Eur J Biochem* **87**, 525-531, doi:DOI 10.1111/j.1432-1033.1978.tb12403.x (1978).
83 Shanmughapriya, S., Kiran, G. S., Selvin, J., Thomas, T. A. & Rani, C. Optimization, purification, and characterization of extracellular mesophilic alkaline cellulase from sponge-associated *Marinobacter* sp MSI032. *Appl Biochem Biotech* **162**, 625-640, doi:10.1007/s12010-009-8747-0 (2010).
84 Wang, C. Y. *et al.* Purification and characterization of a novel halostable cellulase from *Salinivibrio* sp strain NTU-05. *Enzyme Microb Tech* **44**, 373-379, doi:10.1016/j.enzmictec.2009.02.006 (2009).
85 Chen, P. J., Wei, T. C., Chang, Y. T. & Lin, L. P. Purification and characterization of carboxymethyl cellulase from *Sinorhizobium fredii*. *Bot Bull Acad Sinica* **45**, 111-118 (2004).
86 Stutzenberger, F. J. Cellulolytic activity of *Thermomonospora curvata* - optimal assay conditions, partial purification, and product of cellulase. *Appl Microbiol* **24**, 83-90 (1972).
87 Chararas, C., Eberhard, R., Courtois, J. E. & Petek, F. Purification of 3 cellulases from the xylophageous larvae of *Ergates faber* (*Coleoptera, Cerambycidae*). *Insect Biochem* **13**, 213-218, doi:Doi 10.1016/0020-1790(83)90086-0 (1983).
88 Scrivener, A. M. & Slaytor, M. Properties of the endogenous cellulase from *Panesthia cribrata saussure* and purification of major endo-beta-1,4-glucanase components. *Insect Biochem Molec* **24**, 223-231, doi:Doi 10.1016/0965-1748(94)90001-9 (1994).
89 Sugimura, M., Watanabe, H., Lo, N. & Saito, H. Purification, characterization, cDNA cloning and nucleotide sequencing of a cellulase from the yellow-spotted longicorn beetle, *Psacothea hilaris*. *Eur J Biochem* **270**, 3455-3460, doi:10.1046/j.1432-1033.2003.03735.x (2003).

90 Lew, F. T. & Lewis, L. N. Purification and properties of cellulase from *Phaseolus vulgaris*. *Phytochemistry* **13**, 1359-1366, doi:Doi 10.1016/0031-9422(74)80292-X (1974).
91 Morrison, J. M., Elshahed, M. S. & Youssef, N. H. A multifunctional GH39 glycoside hydrolase from the anaerobic gut fungus *Orpinomyces* sp. strain C1A. *Plos One*, Submitted (2016).
92 Kitpreechavanich, V., Hayashi, M. & Nagai, S. Purification and characterization of extracellular beta-xylosidase and beta-glucosidase from *Aspergillus fumigatus*. *Agr Biol Chem* **50**, 1703-1711 (1986).
93 John, M., Schmidt, B. & Schmidt, J. Purification and some properties of five endo-1,4-beta-D-xylanases and a beta-D-xylosidase produced by a strain of *Aspergillus niger*. *Can J Biochem Cell B* **57**, 125-134 (1979).
94 vanPeij, N. N. M. E., Brinkmann, J., Vrsanska, M., Visser, J. & deGraaff, L. H. Beta-xylosidase activity, encoded by xlnD, is essential for complete hydrolysis of xylan by *Aspergillus niger* but not for induction of the xylanolytic enzyme spectrum. *Eur J Biochem* **245**, 164-173 (1997).
95 Hayashi, S., Ohno, T., Ito, M. & Yokoi, H. Purification and properties of the cell-associated ss-xylosidase from *Aureobasidium*. *J Ind Microbiol Biot* **26**, 276-279, doi:DOI 10.1038/sj.jim.7000120 (2001).
96 Garcia-Campayo, V. & Wood, T. M. Purification and characterization of a beta-D-xylosidase from the anaerobic rumen fungus *Neocallimastix frontalis*. *Carbohyd Res* **242**, 229-245, doi:Doi 10.1016/0008-6215(93)80037-F (1993).
97 Wilson, C. A., Mccrae, S. I. & Wood, T. M. Characterization of a beta-D-glucosidase from the anaerobic rumen fungus *Neocallimastix frontalis* with particular reference to attack on cello-oligosaccharides. *J Biotechnol* **37**, 217-227, doi:Doi 10.1016/0168-1656(94)90129-5 (1994).
98 Hebraud, M. & Fevre, M. Characterization of Glycoside and Polysaccharide Hydrolases Secreted by the Rumen Anaerobic Fungi Neocallimastix-Frontalis, Sphaeromonas-Communis and Piromonas-Communis. *J Gen Microbiol* **134**, 1123-1129 (1988).
99 Chen, H. Z., Li, X. L. & Ljungdahl, L. G. Isolation and properties of an extracellular beta-glucosidase from the polycentric rumen fungus *Orpinomyces s*p strain pc-2. *Appl Environ Microbiol* **60**, 64-70 (1994).
100 Harhangi, H. R. *et al.* A highly expressed family 1 beta-glucosidase with transglycosylation capacity from the anaerobic fungus *Piromyces* sp E2. *BBA-Gene Struct Expr* **1574**, 293-303, doi:Pii S0167-4781(01)00380-3
Doi 10.1016/S0167-4781(01)00380-3 (2002).
101 Meyer, H. P. & Canevascini, G. Separation and some properties of 2 intracellular beta-glucosidases of *Sporotrichum* (*Chrysosporium*) thermophile. *Appl Environ Microbiol* **41**, 924-931 (1981).
102 Parry, N. J. *et al.* Biochemical characterization and mechanism of action of a thermostable beta-glucosidase purified from *Thermoascus aurantiacus*. *Biochem J* **353**, 117-127 (2001).
103 Ximenes, F. D., Silveira, F. Q. D. & Ximenes, E. Production of beta-xylosidase activity by *Trichoderma harzianum* strains. *Curr Microbiol* **33**, 71-77, doi:DOI 10.1007/s002849900077 (1996).

104 Kormelink, F. J. M., Searlevanleeuwen, M. J. E., Wood, T. M. & Voragen, A. G. J. Purification and characterization of 3 endo-(1,4)-beta-xylanases and one beta-xylosidase from *Aspergillus awamori*. *J Biotechnol* **27**, 249-265, doi:Doi 10.1016/0168-1656(93)90089-6 (1993).
105 Kiss, T. & Kiss, L. Purification and characterization of an extracellular beta-D-xylosidase from *Aspergillus carbonarius*. *World J Microb Biot* **16**, 465-470, doi:Doi 10.1023/A:1008912025896 (2000).
106 Kumar, S. & Ramon, D. Purification and regulation of the synthesis of a beta-xylosidase from *Aspergillus nidulans*. *FEMS Microbiol Lett* **135**, 287-293, doi:DOI 10.1111/j.1574-6968.1996.tb08003.x (1996).
107 Rizzatti, A. C. S., Jorge, J. A., Terenzi, H. F., Rechia, C. G. V. & Polizeli, M. L. T. M. Purification and properties of a thermostable extracellular beta-D-xylosidase produced by a thermotolerant Aspergillus phoenicis. *J Ind Microbiol Biot* **26**, 156-160, doi:DOI 10.1038/sj.jim.7000107 (2001).
108 Sulistyo, J., Kamiyama, Y. & Yasui, T. Purification and some properties of *Aspergillus pulverulentus* beta-xylosidase with transxylosylation capacity. *J Ferment Bioeng* **79**, 17-22, doi:Doi 10.1016/0922-338x(95)92737-W (1995).
109 Dobberstein, J. & Emeis, C. C. Purification and characterization of beta-xylosidase from *Aureobasidium pullulans*. *Appl Microbiol Biot* **35**, 210-215 (1991).
110 Saha, B. C. Purification and properties of an extracellular beta-xylosidase from a newly isolated *Fusarium proliferatum*. *Bioresource Technol* **90**, 33-38, doi:10.1016/S0960-8524(03)00098-1 (2003).
111 Saha, B. C. Purification and characterization of an extracellular beta-xylosidase from a newly isolated *Fusarium verticillioides*. *J Ind Microbiol Biot* **27**, 241-245, doi:DOI 10.1038/sj.jim.7000184 (2001).
112 Iembo, T., Azevedo, M. O., Bloch, C. & Filho, E. X. F. Purification and partial characterization omicron f a new beta-xylosidase from *Humicola grisea var. thermoidea*. *World J Microb Biot* **22**, 475-479, doi:10.1007/s11274-005-9059-3 (2006).
113 Hebraud, M. & Fevre, M. Purification and characterization of an extracellular beta-xylosidase from the rumen anaerobic fungus *Neocallimastix frontalis*. *FEMS Microbiol Lett* **72**, 11-16, doi:DOI 10.1111/j.1574-6968.1990.tb03853.x (1990).
114 Zhu, H., Cheng, K. J. & Forsberg, C. W. A truncated beta-xylosidase from the anaerobifungus *Neocallimastix patriciarum* 27. *Can J Microbiol* **40**, 484-490 (1994).
115 Deshpande, V., Lachke, A., Mishra, C., Keskar, S. & Rao, M. Mode of action and properties of xylanase and beta-xylosidase from *Neurospora crassa*. *Biotechnol Bioeng* **28**, 1832-1837, doi:DOI 10.1002/bit.260281210 (1986).
116 Matsuo, M., Fujie, A., Win, M. & Yasui, T. 4 types of beta-xylosidases from *Penicillium wortmanni* ifo-7237. *Agr Biol Chem* **51**, 2367-2379 (1987).
117 Tuohy, M. G., Puls, J., Claeyssens, M., Vrsanska, M. & Coughlan, M. P. The xylan-degrading enzyme-system of *Talaromyces emersonii* - novel enzymes with activity against aryl beta-D-xylosides and unsubstituted xylans. *Biochem J* **290**, 515-523 (1993).
118 Poutanen, K. & Puls, J. Characteristics of *Trichoderma reesei* beta-xylosidase and its use in the hydrolysis of solubilized xylans. *Appl Microbiol Biot* **28**, 425-432 (1988).
119 Matsuo, M. & Yasui, T. Purification and some properties of beta-xylosidase from *Trichoderma viride*. *Agr Biol Chem* **48**, 1845-1852 (1984).

120 Wagschal, K., Franqui-Espiet, D., Lee, C. C., Robertson, G. H. & Wong, D. W. S. Cloning, expression and characterization of a glycoside hydrolase family 39 xylosidase from Bacillus halodurans C-125. *Appl Biochem Biotech* **146**, 69-78, doi:10.1007/s12010-007-8055-5 (2008).
121 Nanmori, T., Watanabe, T., Shinke, R., Kohno, A. & Kawamura, Y. Purification and properties of thermostable xylanase and beta-xylosidase produced by a newly isolated *Bacillus stearothermophilus* strain. *J Bacteriol* **172**, 6669-6672 (1990).
122 Lama, L., Calandrelli, V., Gambacorta, A. & Nicolaus, B. Purification and characterization of thermostable xylanase and beta-xylosidase by the thermophilic bacterium *Bacillus thermantarcticus*. *Res Microbiol* **155**, 283-289, doi:10.1016/j.resmic.2004.02.001 (2004).
123 Utt, E. A., Eddy, C. K., Keshav, K. F. & Ingram, L. O. Sequencing and expression of the *Butyrivibrio fibrisolvens* xylb gene encoding a novel bifunctional protein with beta-D-xylosidase and alpha-L-arabinofuranosidase activities. *Appl Environ Microbiol* **57**, 1227-1234 (1991).
124 Correa, J. M. *et al.* Expression and characterization of a GH39 beta-xylosidase II from *Caulobacter crescentus*. *Appl Biochem Biotech* **168**, 2218-2229, doi:10.1007/s12010-012-9931-1 (2012).
125 Sakka, K. *et al.* Nucleotide sequence of the *Clostridium stercorarium* xyla gene encoding a bifunctional protein with beta-D-xylosidase and alpha-L-arabinofuranosidase activities, and properties of the translated product. *Biosci Biotech Bioch* **57**, 268-272 (1993).
126 Bhalla, A., Bischoff, K. M. & Sani, R. K. Highly thermostable GH39 beta-xylosidase from a *Geobacillus* sp strain WSUCF1. *BMC Biotechnol* **14**, 963-973, doi:Artn 963
10.1186/S12896-014-0106-8 (2014).
127 Czjzek, M. *et al.* Enzyme-substrate complex structures of a GH39 beta-xylosidase from *Geobacillus stearothermophilus*. *J Mol Biol* **353**, 838-846, doi:10.1016/j.jmb.2005.09.003 (2005).
128 Shao, W. L. *et al.* Characterization of a bovel beta-xylosidase, XylC, from *Thermoanaerobacterium saccharolyticum* JW/SL-YS485. *Appl Environ Microbiol* **77**, 719-726, doi:10.1128/Aem.01511-10 (2011).
129 Shao, W. L. & Wiegel, J. Purification and characterization of a thermostable beta-xylosidase from *Thermoanaerobacter ethanolicus*. *J Bacteriol* **174**, 5848-5853 (1992).
130 Lee, R. C., Hrmova, M., Burton, R. A., Lahnstein, J. & Fincher, G. B. Bifunctional family 3 glycoside hydrolases from barley with alpha-L-arabinofuranosidase and beta-D-xylosidase activity - characterization, primary structures, and COOH-terminal processing. *J Biol Chem* **278**, 5377-5387, doi:10.1074/jbc.M210627200 (2003).
131 Cardoso, O. A. V. & Ferreira, E. X. Purification and characterization of a novel cellulase-free xylanase from *Acrophialophora nainiana*. *FEMS Microbiol Lett* **223**, 309-314, doi:10.1016/S0378-1097(03)00392-6 (2003).
132 Salles, B. C., Cunha, R. B., Fontes, W., Sousa, M. V. & Filho, E. X. F. Purification and characterization of a new xylanase from *Acrophialophora nainiana*. *J Biotechnol* **81**, 199-204, doi:Doi 10.1016/S0168-1656(00)00280-7 (2000).
133 Ximenes, F. D., de Sousa, M. V., Puls, J., da Silva, F. G. & Ferreira, E. X. Purification and characterization of a low-molecular-weight xylanase produced by *Acrophialophora nainiana*. *Curr Microbiol* **38**, 18-21, doi:Doi 10.1007/Pl00006765 (1999).

134 Lucena-Neto, S. D. & Ferreira, E. X. Purification and characterization of a new xylanase from *Humicola grisea var. Thermoidea*. *Braz J Microbiol* **35**, 86-90, doi:Doi 10.1590/S1517-83822004000100014 (2004).
135 Monti, R., Terenzi, H. F. & Jorge, J. A. Purification and properties of an extracellular xylanase from the thermophilic fungus *Humicola grisea var thermoidea*. *Can J Microbiol* **37**, 675-681 (1991).
136 Hebraud, M. & Fevre, M. Characterization of glycoside and polysaccharide hydrolases secreted by the rumen anaerobic fungi *Neocallimastix frontalis*, *Sphaeromonas communis* and *Piromonas communis*. *J Gen Microbiol* **134**, 1123-1129 (1988).
137 Li, L. T., Tian, H. M., Cheng, Y. Q., Jiang, Z. Q. & Yang, S. Q. Purification and characterization of a thermostable cellulase-free xylanase from the newly isolated *Paecilomyces themophila*. *Enzyme Microb Tech* **38**, 780-787, doi:10.1016/j.enzmictec.2005.08.007 (2006).
138 Dutta, T. *et al.* A novel cellulase free alkaliphilic xylanase from alkali tolerant *Penicillium citrinum*: production, purification and characterization. *Lett Appl Microbiol* **44**, 206-211, doi:10.1111/j.1472-765X.2006.02042.x (2007).
139 Silveira, F. Q. D. *et al.* A new xylanase from a *Trichoderma harzianum* strain. *J Ind Microbiol Biot* **23**, 682-685 (1999).
140 Tan, L. U. L., Yu, E. K. C., Louisseize, G. W. & Saddler, J. N. Inexpensive, rapid procedure for bulk purification of cellulase-free beta-1,4-D-xylanase of high specific activity. *Biotechnol Bioeng* **30**, 96-100, doi:DOI 10.1002/bit.260300114 (1987).
141 Dhillon, A., Gupta, J. K. & Khanna, S. Enhanced production, purification and characterisation of a novel cellulase-poor thermostable, alkalitolerant xylanase from *Bacillus circulans* AB 16. *Process Biochem* **35**, 849-856, doi:Doi 10.1016/S0032-9592(99)00152-1 (2000).
142 Archana, A. & Satyanarayana, T. Purification and characterization of a cellulase-free xylanase of a moderate thermophile *Bacillus licheniformis* A99. *World J Microb Biot* **19**, 53-57, doi:Doi 10.1023/A:1022527702400 (2003).
143 Morosoli, R., Bertrand, J. L., Mondou, F., Shareck, F. & Kluepfel, D. Purification and properties of a xylanase from *Streptomyces lividans*. *Biochem J* **239**, 587-592 (1986).
144 Li, X. T. *et al.* Purification and characterization of a cellulase-free, thermostable xylanase from *Streptomyces rameus* L2001 and its biobleaching effect on wheat straw pulp. *Biochem Eng J* **52**, 71-78, doi:10.1016/j.bej.2010.07.006 (2010).
145 Tsujibo, H. *et al.* Purification, properties, and partial amino-acid-sequences of thermostable xylanases from *Streptomyces thermoviolaceus* opc-520. *Appl Environ Microbiol* **58**, 371-375 (1992).
